# Neutrophil-derived IL-1β Is Sufficient for Abscess Formation in Immunity against *Staphylococcus aureus* in Mice

**DOI:** 10.1371/journal.ppat.1003047

**Published:** 2012-11-29

**Authors:** John S. Cho, Yi Guo, Romela Irene Ramos, Frank Hebroni, Seema B. Plaisier, Caiyun Xuan, Jennifer L. Granick, Hironori Matsushima, Akira Takashima, Yoichiro Iwakura, Ambrose L. Cheung, Genhong Cheng, Delphine J. Lee, Scott I. Simon, Lloyd S. Miller

**Affiliations:** 1 Department of Medicine, Division of Dermatology, David Geffen School of Medicine at University of California Los Angeles (UCLA), Los Angeles, California, United States of America; 2 Department of Translational Immunology, Dirks/Dougherty Laboratory for Cancer Research, John Wayne Cancer Institute, Santa Monica, California, United States of America; 3 Department of Biomedical Engineering, University of California Davis, Davis, California, United States of America; 4 Department of Medical Microbiology and Immunology, University of Toledo College of Medicine, Toledo, Ohio, United States of America; 5 Center for Experimental Medicine and Systems Biology, Institute of Medical Science, University of Tokyo, Minato-ku, Tokyo, Japan and Core Research for Evolutional Science and Technology (CREST), Japan Science and Technology Agency, Saitama, Japan; 6 Department of Microbiology and Immunology, Dartmouth Medical School, Hanover, New Hampshire, United States of America; 7 Department of Microbiology, Immunology, and Molecular Genetics, David Geffen School of Medicine at University of California Los Angeles (UCLA), Los Angeles, California, United States of America; 8 Department of Dermatology, Johns Hopkins University School of Medicine, Baltimore, Maryland, United States of America; National Institute of Allergy and Infectious Diseases, National Institutes of Health, United States of America

## Abstract

Neutrophil abscess formation is critical in innate immunity against many pathogens. Here, the mechanism of neutrophil abscess formation was investigated using a mouse model of *Staphylococcus aureus* cutaneous infection. Gene expression analysis and *in vivo* multispectral noninvasive imaging during the *S. aureus* infection revealed a strong functional and temporal association between neutrophil recruitment and IL-1β/IL-1R activation. Unexpectedly, neutrophils but not monocytes/macrophages or other MHCII-expressing antigen presenting cells were the predominant source of IL-1β at the site of infection. Furthermore, neutrophil-derived IL-1β was essential for host defense since adoptive transfer of IL-1β-expressing neutrophils was sufficient to restore the impaired neutrophil abscess formation in *S. aureus*-infected IL-1β-deficient mice. *S. aureus*-induced IL-1β production by neutrophils required TLR2, NOD2, FPR1 and the ASC/NLRP3 inflammasome in an α-toxin-dependent mechanism. Taken together, IL-1β and neutrophil abscess formation during an infection are functionally, temporally and spatially linked as a consequence of direct IL-1β production by neutrophils.

## Author Summary

Invasive infections caused by the human pathogen *Staphylococcus aureus* result in more deaths annually than infections caused by any other single infectious agent in the United States. Although neutrophil recruitment and abscess formation is crucial for effective host defense against this pathogen, how neutrophils sense and mount an inflammatory response are not completely clear. Using gene expression analysis and *in vivo* bioluminescence and fluorescence imaging, we found that neutrophil recruitment during a *S. aureus* cutaneous infection is functionally and temporally linked to IL-1β/IL-1R activation. Surprisingly, neutrophils themselves were determined to be the most abundant cell type that produced IL-1β during infection. Further, neutrophil-derived IL-1β, in the absence of other cellular sources of IL-1β, was sufficient for neutrophil recruitment, abscess formation, and bacterial clearance. Finally, mouse neutrophils produced IL-1β in direct response to live *S. aureus in vitro*. These findings expand our understanding of the acute neutrophil response to infection in which early recruited neutrophils serve as a source of IL-1β that is essential for amplifying and sustaining the neutrophilic response to promote abscess formation and bacterial clearance. Therapies aimed at promoting IL-1β production by neutrophils may be an effective immunotherapeutic strategy to control *S. aureus* infections.

## Introduction

Neutrophil abscess formation represents an important component of the innate immune response, which helps control the spread of an invading pathogen into deeper tissues and systemically [1]. At the site of infection, neutrophils primarily function through the phagocytosis of microorganisms and utilize a variety of antimicrobial mechanisms to mediate pathogen killing [2].

To investigate mechanisms that promote neutrophil recruitment and abscess formation, we chose to use *S. aureus* cutaneous infection as a model [3]. This gram-positive extracellular bacterium is responsible for the vast majority of skin and soft tissue infections in humans and is a common cause of invasive and often life-threatening infections such as bacteremia, abscesses of various organs, septic arthritis, osteomyelitis, endocarditis, pneumonia and sepsis [4], [5]. *S. aureus* infection serves as an excellent model system to study neutrophil recruitment since neutrophil abscess formation is required for bacterial clearance in a variety of mouse models of *S. aureus* infection, including cutaneous infection, bacteremia, septic arthritis and brain abscesses [6]–[8]. The critical role of neutrophils in host defense against *S. aureus* is also seen in humans, since patients with genetic or acquired conditions with defective neutrophil number or function suffer from recurrent and invasive *S. aureus* infections in various tissues and organs, including the skin [9].

It is well established that IL-1β plays a central role in initiating the neutrophilic response against *S. aureus* infections [7], [10], [11]. This is mediated by IL-1β activation of IL-1R/MyD88 signaling, which triggers NF-κB and other signaling molecules that induce proinflammatory mediators and chemokines to promote neutrophil trafficking from the circulation into the infected tissue [9]. Given this essential function of IL-1β, there has been intense interest in understanding how its production is triggered during an infection *in vivo*. On a cellular level, two different signals are required for IL-1β production in response to *S. aureus*. The first is production of pro-IL-1β, which is in part mediated by activation of pattern recognition receptors (PRRs) such as TLR2, a cell surface PRR that recognizes *S. aureus* lipopeptides and lipoteichoic acid [12], [13], and NOD2, a cytoplasmic PRR that recognizes muramyl dipeptide, which is a breakdown product of *S. aureus* peptidoglycan [14], [15]. The second signal is the triggering of the NLRP3 inflammasome to induce caspase-1 activation and subsequent cleavage of pro-IL-1β into mature IL-1β, the active and secreted cytokine [16]–[18].

The mechanism for inducing IL-1β-dependent neutrophil recruitment in infected tissues *in vivo* is complex and involves interactions among epithelial cells, stromal cells, resident immune cells, endothelial cells and recruited immune cells. It is known that IL-1β produced at the site of *S. aureus* skin infection promotes neutrophil recruitment by inducing neutrophil-attracting chemokines and granulopoiesis factors directly via activating IL-1R/MyD88-signaling and indirectly through the production of IL-17 by T cells [3], [11], [19]. A key question is which cell types are responsible for IL-1β production during a *S. aureus* infection *in vivo* and how these cells utilize PRRs and the inflammasome to induce its production. The precise mechanism is particularly relevant since many different cells, including keratinocytes, mast cells, Langerhans cells, dendritic cells and monocytes/macrophages, can produce IL-1β in various *in vivo* models of skin inflammation and infection [20]–[23]. In the current study, we used gene expression analysis and noninvasive *in vivo* imaging to determine the functional and temporal kinetics, cellular sources and mechanisms by which IL-1β induces neutrophil abscess formation during a *S. aureus* skin infection.

## Results

### Gene Expression Analysis of a *S. aureus* Skin Infection

**Figure 1:**
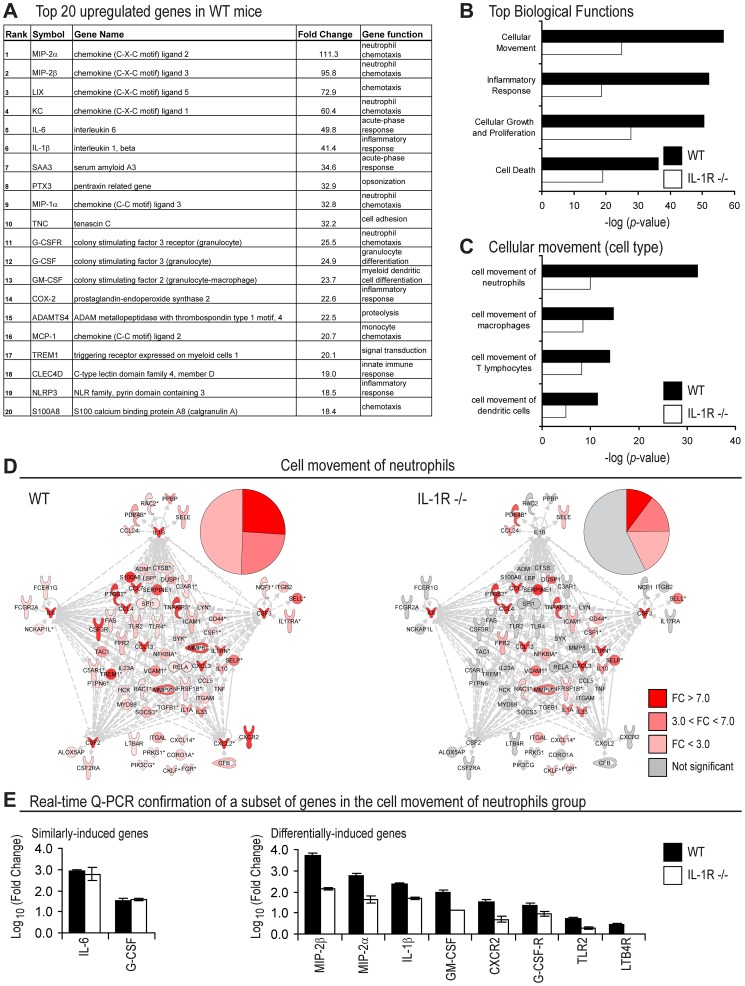
Gene expression analysis in wt and IL-1R-deficient mice after *S. aureus* skin infection. Wt and IL-1R^−/−^ mice were inoculated intradermally with *S. aureus* and gene expression analysis was performed on skin specimens taken at 4 hrs and compared with samples from uninfected skin (n = 3 mice per group). Upregulated genes were defined according to the criteria of fold-change (FC) >1.5, p-value<0.05. (A) Top 20 induced genes in wt mice ordered by FC (gene function was assigned using NetAffx Gene Ontology Mining Tool [Affymetrix]). (B) Top four biological functions in wt versus IL-1R^−/−^ mice generated by Ingenuity Pathway analysis (ranked by p-value). (C) Top four cell types in the Cellular Movement functional group (ranked by p-value). (D) Functional network analysis of genes in the Cell Movement of Neutrophils group in wt mice (left) compared with the same genes in IL-1R^−/−^ mice (right). The genes in the networks and pie charts are shaded according to FC. Red: >7.0 FC (strongly upregulated), pink: 3.0<FC<7.0 (moderately upregulated), light pink: <3.0 FC (slightly upregulated), or grey: no change or not statistically significant. (E) Real-time Q-PCR confirmation (mean log_10_ fold change ± SEM) of 2 representative genes that were similarly-induced between wt and IL-1R-deficient mice and 8 representative genes that were differentially-induced in wt mice compared with IL-1R-deficient mice from the Cell Movement of Neutrophils sub-group from microarray analysis in Fig. 1D.

Mice deficient in IL-1β, IL-1R or MyD88, but not IL-1α, exhibit a severe impairment in neutrophil abscess formation at the site of infection in this mouse model of *S. aureus* intradermal infection [3], [11], indicating that IL-1β is the major cytokine that initiates the IL-1R/MyD88-dependent pathway for neutrophil recruitment. Given these results and evidence from humans and mice that neutrophils are essential for clearance of *S. aureus* infections [9], gene expression analysis was performed in an attempt to link IL-1β/IL-1R-dependent gene induction with neutrophil recruitment. To accomplish this, we used a model of *S. aureus* cutaneous infection in mice, which involves intradermal inoculation of *S. aureus* (2×10^6^ CFUs of strain SH1000) in the dorsal back skin of mice [3]. Gene expression analysis was first performed on skin biopsy samples from wt and IL-1R-deficient mice at 4 hrs after *S. aureus* infection and from uninfected skin. This time point was chosen because we previously observed substantially decreased IL-1β protein levels in *S. aureus*-infected skin of IL-1R-deficient mice compared with wt mice at 6 hrs after infection [3] and the difference in mRNA levels of IL-1β likely preceded the changes in IL-1β protein levels. By using the criteria that upregulated genes were >1.5-fold higher (p-value<0.05) than baseline, there were 1,288 genes upregulated in wt mice and 606 genes upregulated in IL-1R-deficient mice (Fig. S1). Comparing *S. aureus*-infected skin of wt mice versus uninfected skin, the top 4 induced genes were neutrophil-attracting CXC chemokines, including *CXCL1* (*KC*), *CXCL2* (*MIP2α*), *CXCL3* (*MIP2β*) and *CXCL5* (*LIX*) (Fig. 1A), which bind to CXCR2 on mouse neutrophils to induce chemotaxis [24]. The neutrophil granulopoiesis factors *G-CSF* and *GM-CSF*, as well as the proinflammatory cytokines *IL-1β*, *IL-6* and the inflammasome component *NLRP3*, were also among the top 20 genes. These data indicate that many of the most highly induced genes in *S. aureus*-infected wt mice were associated with neutrophil chemotaxis, granulopoiesis and IL-1β production.

Pathway analysis (Ingenuity) was then used to categorize the genes upregulated in *S. aureus*-infected wt versus IL-1R-deficient mice into functional groups. In wt mice, the most significantly upregulated functional pathway was the Cellular Movement group, which included 354 genes (p = 3×10^−57^) (Fig. 1B). In contrast, in IL-1R-deficient mice, the Cellular Movement functional group included only 256 genes and had lower statistical significance (p = 2×10^−25^). Categorization of the Cellular Movement functional group by cell type revealed that in wt mice, the Cell Movement of Neutrophils sub-group included 77 genes and was the most statistically significant (p = 7×10^−33^). Neutrophils were followed in order of number of genes and significance by macrophages (41 genes; p = 2×10^−15^), T cells (41 genes; p = 9×10^−15^) and dendritic cells (25 genes; p = 3×10^−12^). In contrast, in IL-1R-deficient mice, the categorization of the Cell Movement group by cell type was radically different with less numbers of upregulated genes. These differences were most evident for neutrophils, (44 genes; p = 1×10^−10^), followed by macrophages (28 genes; p = 4×10^−9^), T cells (29 genes; p = 7×10^−9^) and dendritic cells (10 genes; p = 1×10^−5^).

The level of induction of upregulated genes in the Cell Movement of Neutrophils group was then compared between wt and IL-1R-deficient mice using functional network analysis (Fig. 1D). All 77 genes associated with Cell Movement of Neutrophils were significantly upregulated in wt mice. In contrast, over half (45 of the 77 genes), including *CXCL2*, *GM-CSF* and *IL-1β*, were not significantly induced in IL-1R-deficient mice. To confirm these results, the expression of a subset of genes of interest in the Cell Movement of Neutrophils sub-group (Fig. 1D) were evaluated by quantitative real-time PCR (Q-PCR), which included 2 genes that were similarly-induced (*IL-6* and *G-CSF*) and 8 genes that were differentially-induced in wt and IL-1R-deficient mice (*MIP-2β*, *MIP-2α*, *IL-1β*, *GM-CSF*, *CXCR2*, *G-CSF-R*, *TLR2* and *LTB4R*) (Fig. 1E). In agreement with the microarray data (Fig. 1D), we found that the expression of *IL-6* and *G-CSF* between wt and IL-1R deficient mice was similar and the differentially-induced genes had higher expression in wt mice compared with IL-1R-deficient mice. Taken together, these results indicate that genes associated with neutrophil recruitment were upregulated in response to *S. aureus* skin infection and that the induction of these genes was largely dependent on IL-1β/IL-1R signaling.

To differentiate between the specific anti-*S. aureus* response versus the response to a live infection, the skin of wt and IL-1R-deficient mice was inoculated with either live or heat-killed *S. aureus* and Q-PCR was performed on skin samples taken at 4 hrs after inoculation and from uninfected mice (Fig. S2). We found that the top six genes on the microarray were highly expressed in skin samples infected with live *S. aureus* (ranging from 242- to 5696-fold) or heat-killed *S. aureus* (ranging from 46- to 1706-fold); however, the level of induction with heat-killed *S. aureus* was generally a magnitude less (decreased from 4.5-fold to 14.1-fold) than live *S. aureus*. To determine whether the pattern of gene induction in wt and IL-1R-deficient mice was similar or different in response to live versus heat-killed *S. aureus*, the same subset of genes of interest in the Cell Movement of Neutrophils sub-group in Fig. 1E was compared (Fig. S2B, C). Live and heat-killed *S. aureus* had similar expression of *IL-6* and *G-CSF* between wt and IL-1R deficient mice and the differentially-induced genes had higher expression in wt mice compared with IL-1R-deficient mice. However, as with the top 6 induced genes (Fig. S2A), the levels of induction of these genes were lower with heat-killed *S. aureus* compared with live *S. aureus*. In summary, the difference in gene expression patterns between wt and IL-1R-deficient mice was consistent between live and heat-killed *S. aureus* (albeit the live *S. aureus* resulted in higher gene expression than heat-killed *S. aureus*), demonstrating that the immune response is more intense in the presence of the live bacterial infection. These results suggest that *S. aureus*-specific immune responses are significantly impaired in IL-1R-deficient mice. However, we cannot exclude the possibility that the impaired immune responses in the IL-1R-deficient mice were due to a dysregulated immune response in IL-1R-deficient mice in which any stimulus would elicit an aberrant response with a similar pattern of gene expression.

### *In vivo* Kinetics of IL-1β Production and Neutrophil Recruitment

Although IL-1β is the major cytokine that induces IL-1R/MyD88-dependent neutrophil recruitment during a *S. aureus* skin infection [11], the source and kinetics of IL-1β production during an infection *in vivo* has remained unknown. Therefore, advanced techniques of *in vivo* bioluminescence and fluorescence imaging were combined to provide an approximation of the kinetics of IL-1β production and neutrophil recruitment longitudinally over the time course of the *S. aureus* cutaneous infection. This was accomplished by performing the intradermal inoculation of a bioluminescent *S. aureus* strain in two fluorescence reporter mouse strains: (1) pIL1-DsRed transgenic mice, which express the red fluorescent protein DsRed under the control of the mouse IL-1β promoter [21], and (2) LysEGFP mice, which possess green fluorescent myeloid cells (mostly neutrophils) due to a knock-in of the EGFP gene into the lysozyme M locus [25]. *In vivo* noninvasive whole animal imaging was then used to track the *S. aureus* bacterial burden while simultaneously monitoring IL-1β production or neutrophil recruitment in the same anesthetized mice over the 14 day course of infection. Advantages and limitations of using this strategy of *in vivo* imaging to quantify these endpoints are described in the Discussion.

**Figure 2:**
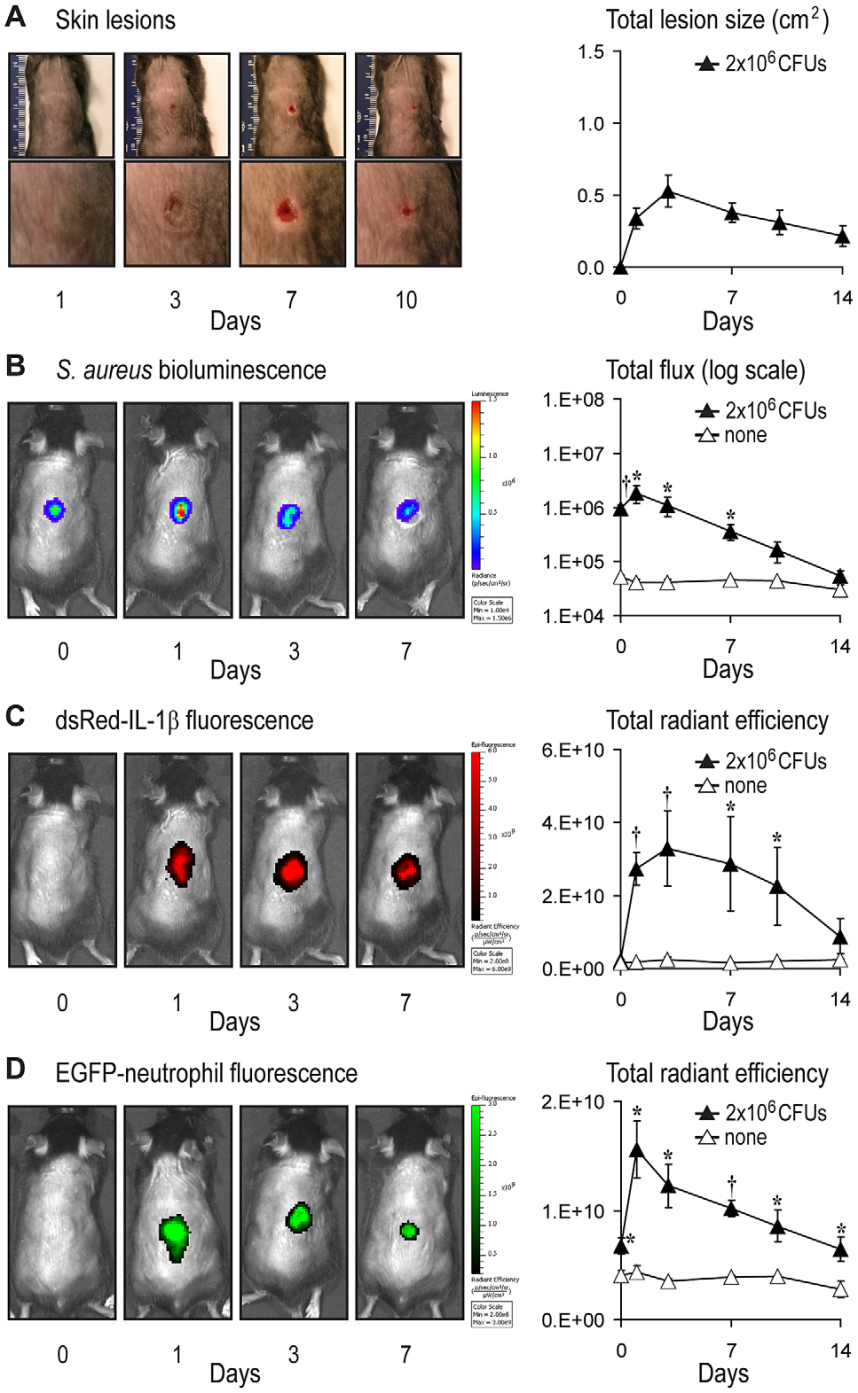
Real-time kinetics of IL-1β production and neutrophil recruitment after *S. aureus* skin infection. pIL1-DsRed reporter mice or LysEGFP mice were inoculated intradermally with *S. aureus*. (A) Representative skin lesions (left) (entire dorsal backs [top, millimeter ruler shown for scale] and close-ups of lesions [bottom]) and mean total lesion size (cm^2^) ± SEM (right). (B) Representative *in vivo S. aureus* bioluminescence (left) and mean total flux (photons/s) ± SEM (logarithmic scale) (right). (C) Representative *in vivo* EGFP-neutrophil fluorescence (left) and mean total radiant efficiency (photons/s)/(µW/cm^2^) ± SEM (right). (D) Representative *in vivo* DsRed-IL-1β fluorescence (left) and mean total radiant efficiency (photons/s)/(µW/cm^2^) ± SEM (right). Data are from 2 experiments with at least 4 mice/group per experiment. *p<0.05; ^†^p<0.01, *S. aureus*-infected mice versus none (sham injection alone) (Student's *t*-test).

Infection with *S. aureus* resulted in the development of visible skin lesions, which had a maximum size of 0.53±0.11 cm^2^ by day 3, and healed by day 14 (Fig. 2A). *In vivo* bioluminescence signals, which closely estimate the bacterial CFUs harvested from the skin lesions during infection [3], [19], peaked on day 1 (up to 1.8±0.7×10^6^ photons/s) and slowly decreased to background levels by day 14 (Fig. 2B). IL-1β-DsRed and EGFP-neutrophil fluorescence signals were significantly higher than uninfected control mice at all time points, peaking on days 3 and 1, respectively (up to 2.7±0.5×10^10^ and 1.6±0.3×10^10^ [photons/s]/[µW/cm^2^], respectively) and decreased to background levels by day 14 (Fig. 2C). In summary, IL-1β-DsRed fluorescence and EGFP-neutrophil fluorescence signals had similar temporal kinetics as they both increased rapidly by day 1 and then decreased along with the *in vivo* bioluminescent signals over the 14 day course of infection.

### The Cellular Sources of IL-1β Induced after Infection

**Figure 3:**
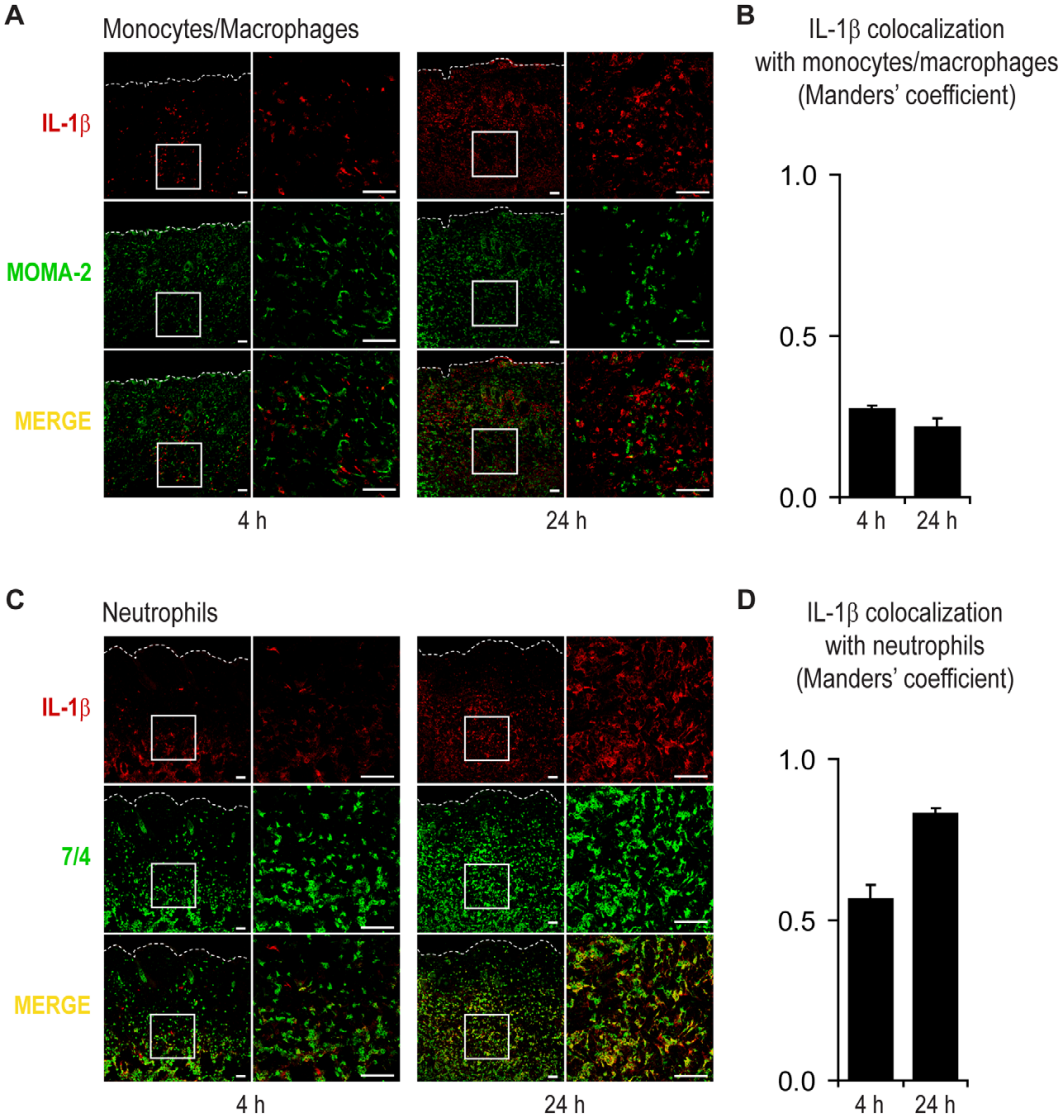
Neutrophils are the predominant cell type that expresses IL-1β after *S. aureus* skin infection. pIL1-DsRed mice were infected intradermally with *S. aureus* and lesional skin specimens were collected at 4 and 24 hrs. Representative photomicrographs of sections labeled with anti-DsRed (IL-1β, red) and anti-MOMA2 (monocytes/macrophages, green) (A) or anti-7/4 (neutrophils, green) (C) and sections analyzed by confocal microscopy. Cells expressing both markers appear yellow (merge). High (left) and low (right) magnification images are shown (Scale bars = 50 µm). Dotted line = dermoepidermal junction. Quantification of co-localization of IL-1β-DsRed fluorescence with MOMA2^+^ monocytes/macrophages (B) or 7/4^+^ neutrophils (D) using the Manders' coefficient for a value range of 0 to 1 in which 0 = no pixels co-localize and 1 = all pixels co-localize. Data are representative from 4 mice per group.

Since many different cell types, including keratinocytes, mast cells, Langerhans cells, dendritic cells and macrophages, have the capacity to produce IL-1β in various *in vivo* models of skin inflammation and infection [20]–[23], it is unclear which of these cell types (or potentially other cell types) contribute to IL-1β production during a *S. aureus* skin infection. However, in our previous work using the same *S. aureus* skin infection model in bone marrow chimeric mice, we found that the source of IL-1β was from bone marrow-derived hematopoietic cells because neutrophil recruitment, host-defense and IL-1β production at the site of infection was restored in IL-1β-deficient mice reconstituted with bone marrow from wt mice but not in wt mice reconstituted with bone marrow from IL-1β-deficient mice [11]. Since *in vivo* fluorescence imaging demonstrated that IL-1β production was detected shortly after infection, histological evaluation of skin lesions at 4 and 24 hrs after *S. aureus* skin infection in pIL1-DsRed mice was performed. To identify the number of cells that expressed pro-IL-1β, two-color immunofluorescence labeling and confocal laser microscopy was performed using an antibody against DsRed, which is retained within the cytoplasm of IL-1β-expressing cells [21] in combination with mAbs directed against cell-specific markers. At 4 hrs, the earliest IL-1β-expressing cells were found almost exclusively within the dermis at the site of abscess formation (Fig. 3A, C). To identify these early IL-1β-expressing cells, sections were first co-labeled with anti-DsRed and mAbs directed against CD45 (pan-leukocyte marker) or MHCII (antigen presenting cells) (Fig. S3). The vast majority of IL-1β-expressing cells co-localized with CD45 whereas only a few cells co-localized with MHCII, indicating that antigen presenting cells (e.g. dermal dendritic cells and monocytes/macrophages) were not the predominant cell type that expressed IL-1β. This was somewhat surprising since monocytes/macrophages produce large amounts of IL-1β in response to *S. aureus* or *S. aureus* components *in vitro* [16]–[18]. Since neutrophils represent the majority of cells recruited at early time points to the site the *S. aureus* infection in the skin [3], [11], [19], we next evaluated the expression of IL-1β in monocytes/macrophages and neutrophils using mAbs directed against MOMA2 and 7/4, respectively [26]. We found that only a few IL-1β-expressing cells co-localized with MOMA2 at 4 and 24 hrs after infection (Fig. 3A and S4A). In contrast, the majority of the IL-1β-expressing cells co localized with 7/4 at 4 hrs and especially at 24 hrs after infection (Fig. 3B and S4C).

To quantify the degree of co-localization between IL-1β-expressing cells and the cell-specific markers, image analysis was performed using the Manders' coefficient for a value range of 0 (no pixels co-localize) to 1 (all pixels co-localize) (Fig. 3B,D). The Manders' coefficient between IL-1β-expressing cells and MOMA2^+^ monocytes/macrophages or MHCII^+^ antigen presenting cells at 4 hrs was 0.27 or 0.21, respectively (Fig. 3D and Fig. S4), confirming that these cells represented a minority of IL-1β-expressing cells. In contrast, the Manders' coefficient between the IL-1β-expressing cells and 7/4^+^ neutrophils was 0.56 at 4 hrs and 0.83 at 24 hrs. Although the co-localization of DsRed with the cellular markers does not provide information about how much IL-1β is made per cell, these data suggest that neutrophils represent the most abundant cell type that expresses IL-1β at the site of infection.

Although neutrophils express high levels of 7/4 and monocyte/macrophages express MOMA2 (which includes recently emigrated monocytes and activated macrophages) [26], some subsets of monocytes and macrophages have also been reported to express 7/4 [27]. Therefore, the labeling of 7/4 versus MOMA2 in sections of *S. aureus*-infected mouse skin was compared (Fig. S5). At both 4 and 24 hrs after infection, there was only a rare occasional cell that expressed both 7/4 and MOMA2. Thus, we conclude that the vast majority of the 7/4^+^ and IL-1β-expressing cells were neutrophils.

### Neutrophil-derived IL-1β Is Sufficient for Effective Abscess Formation and Bacterial Clearance

**Figure 4:**
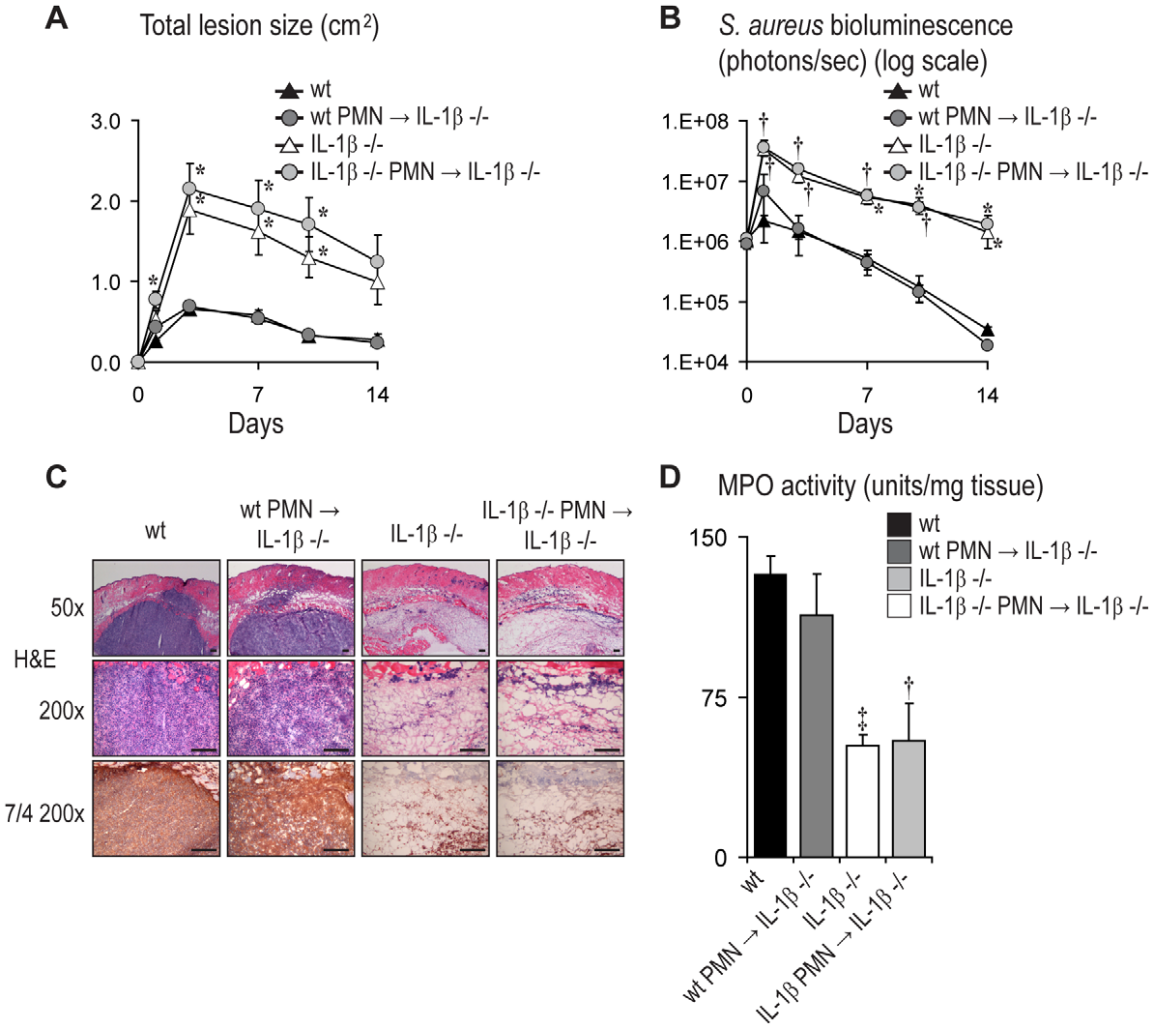
Neutrophil-derived IL-1β is sufficient for abscess formation and bacterial clearance of *S. aureus*-infected mice. Neutrophils from IL-1β^−/−^ or wt donor mice were adoptively transferred into IL-1β^−/−^ recipient mice. After 2 hrs, these mice and normal wt and IL-1β^−/−^ mice were infected intradermally with *S. aureus*. (A) Mean total lesion size (cm^2^) ± SEM. (B) *In vivo* bioluminescence quantified by mean total flux (photons/s) ± SEM (logarithmic scale). (C) Representative photomicrographs of sections labeled with H&E stain and anti-7/4 (neutrophils) (immunoperoxidase method) at 1 day after inoculation. Scale bars = 100 µm. (D) Mean myeloperoxidase (MPO) activity (U/mg tissue weight) ± SEM from lesional skin at 1 day after inoculation. Data are representative from 2 independent experiments with at least 4 mice/group. *p<0.05; ^†^p<0.01, ^‡^p<0.001, IL-1β^−/−^ mice or mice with adoptively transferred neutrophils versus wt mice (Student's *t*-test).

To evaluate the contribution of neutrophil-derived IL-1β in immunity against *S. aureus* skin infection, bone marrow-derived neutrophils isolated by Percoll density gradient centrifugation from wt or IL-1β-deficient donor mice were adoptively transferred into IL-1β-deficient recipient mice (wt PMN→IL-1β^−/−^ mice or IL-1β^−/−^ PMN→IL-1β^−/−^ mice, respectively) (Fig. 4). Two hrs after adoptive transfer, these mice along with normal wt and IL-1β-deficient mice, were inoculated intradermally with *S. aureus*. As expected, IL-1β^−/−^ PMN→IL-1β^−/−^ mice and IL-1β-deficient mice developed larger skin lesions and higher *in vivo* bioluminescence signals compared with wt mice (Fig. 4A and B). The bioluminescent signals could be seen throughout the areas of the infected lesions, indicating that the total lesion size was a reflection of the degree and extent of the bacterial infection. Furthermore, the defects observed in the IL-1β^−/−^ PMN→IL-1β^−/−^ mice or IL-1β-deficient mice were not likely due to impaired neutrophil function in these mice as *in vitro* assays for phagocytosis, degranulation, oxidative burst and bacterial killing were not significantly different between neutrophils from IL-1β-deficient mice and wt mice (Fig. S6). However, wt PMN→IL-1β^−/−^ mice had lesion sizes and *in vivo* bioluminescence signals that were similar to normal wt mice, indicating that neutrophil-derived IL-1β is sufficient for host defense against the *S. aureus* skin infection. To further evaluate if the neutrophils and not other cells (such as the few contaminating monocytes (Fig. S7A) in the adoptively transferred cells played a role in promoting neutrophil recruitment and host defense in the in IL-1β-deficient mice, neutrophils or monocytes were specifically depleted from the adoptively transferred cells by positive selection using anti-Ly6G or anti-CD115 MACS bead separation, respectively (Fig. S7B, C). Depletion of neutrophils reduced the absolute number of transferred Ly6G^+^ neutrophils from 4.6×10^6^ to 1.8×10^6^ neutrophils/mouse (61% depletion efficiency) and did not decrease the absolute number of transferred CD115+ monocytes (∼1.5×10^4^ before and after depletion). Similarly, depletion of monocytes reduced the absolute number of transferred CD115^+^ monocytes from 1.3×10^4^ to 3.0×10^3^ (76.9% depletion efficiency) and did not decrease the absolute number of transferred Ly6G^+^ neutrophils (∼4.6×10^6^ before and after depletion). Although the neutrophil depletion was only 61% complete, the decreased numbers of neutrophils resulted in an inability of adoptively transferred cells from wt mice to rescue the immune impairment in IL-1β-deficient mice. In contrast, monocyte depletion, which decreased the percentage of contaminating monocytes from 0.26% to only 0.06% of the adoptively transferred cells, had no impact on the ability to rescue the immune impairment in IL-1β-deficient mice. These data provide additional evidence that neutrophils and not monocytes in the adoptively transferred cells played a major role in promoting effective neutrophil recruitment and host defense against the cutaneous *S. aureus* infection.

**Figure 5:**
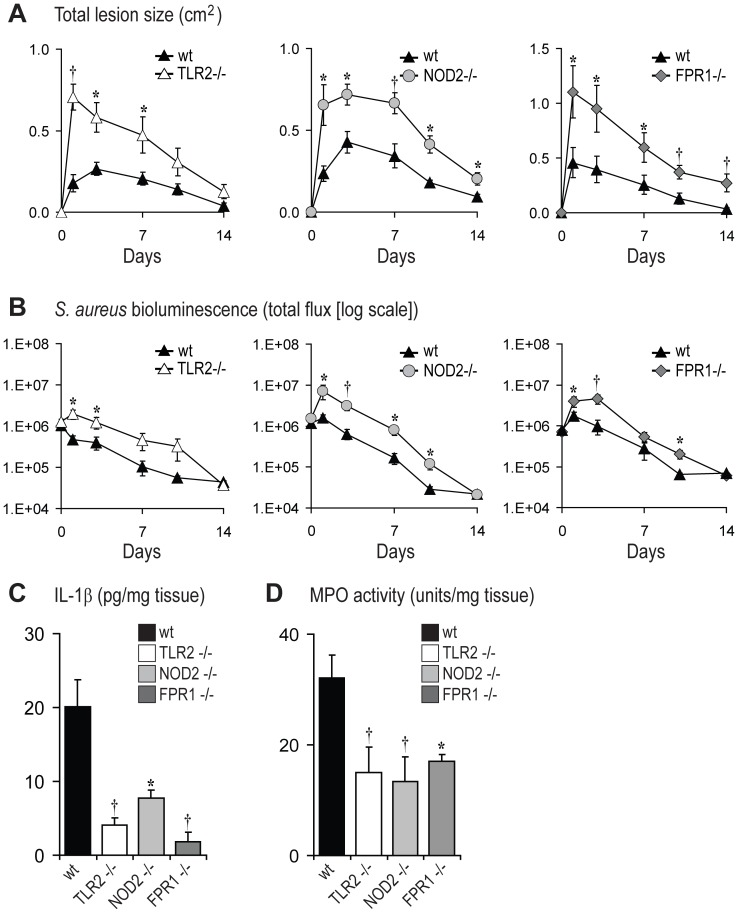
The PRRs TLR2, NOD2, and FPR1 contribute to optimal production of IL-1β during infection. TLR2^−/−^, NOD2^−/−^, FPR1^−/−^ and wt mice were inoculated intradermally with *S. aureus*. (A) Mean total lesion size (cm^2^) ± SEM. (B) *In vivo* bioluminescence quantified by mean total flux (photons/s) ± SEM (logarithmic scale). (C) Mean IL-1β protein levels (pg/mg tissue weight) ± SEM from lesional skin at 4 hrs after inoculation. (D) Mean myeloperoxidase (MPO) activity (U/mg tissue weight) ± SEM from lesional skin at 4 hrs after inoculation. *p<0.05, ^†^p<0.01, TLR2^−/−^, NOD2^−/−^ or FPR1^−/−^ mice versus wt mice (Student's *t*-test). Data are from 2 experiments with at least 4 mice/group per experiment.

Histopathological examination of skin biopsies taken one day after infection with *S. aureus* demonstrated that wt PMN→IL-1β^−/−^ mice and normal wt mice developed large neutrophilic abscesses seen in H&E stained and anti-7/4 labeled sections (Fig. 4C). In contrast, infected skin samples from IL-1β^−/−^ PMN→IL-1β^−/−^ mice and IL-1β-deficient mice had markedly decreased neutrophil recruitment with minimal abscess formation and decreased myeloperoxidase (MPO) activity (which correlates with the degree of neutrophil infiltration) (Fig. 4D). The levels of IL-1β protein expression from infected skin samples at 4 and 24 hrs were evaluated by ELISA and the amount of IL-1β protein at the site of infection in mice adoptively transferred with wt or IL-1β^−/−^ PMN was below the level of detection (data not shown). In contrast, in wt mice at 4 and 24 hrs the levels of IL-1β protein typically exceeded 20 pg/mg tissue weight (Fig. 5C and data not shown). However, immunohistochemistry with an anti-IL-1β mAb identified scattered IL-1β-expressing cells within the abscess at 24 hrs in wt PMN→IL-1β^−/−^ mice but not in IL-1β^−/−^ PMN→IL-1β^−/−^ mice (Fig. S8). These data indicate that adoptively transferred wt neutrophils produced IL-1β at the site of infection and that neutrophil-derived IL-1β is sufficient for promoting effective neutrophil abscess formation and host defense against a cutaneous *S. aureus* infection.

### Production of IL-1β Requires the Expression of PRRs

Certain PRRs have been shown to recognize *S. aureus* components and initiate innate immune responses, including TLR2, a membrane PRR that recognizes *S. aureus* lipopeptides and lipoteichoic acid [12], [13], NOD2, a cytosolic PRR that recognizes muramyl-dipeptide (a breakdown product of *S. aureus* peptidoglycan) [14], [15], and FPRs, which recognize formylated peptides of bacteria [28]. To investigate whether these PRRs contributed to IL-1β production and neutrophil recruitment during a *S. aureus* skin infection *in vivo*, we inoculated wt mice and mice deficient in TLR2, NOD2 or FPR1 with *S. aureus* (Fig. 5). TLR2-, NOD2-, and FPR1-deficient mice all developed larger lesions (up to 4.0-, 2.8- and 2.9-fold, respectively) (Fig. 5A) and higher bioluminescent signals (up to 5.6-, 4.9- and 4.6-fold, respectively) than wt mice (Fig. 5B). Taken together, these results demonstrate that TLR2, NOD2 and FPR1 all significantly contributed to host defense against *S. aureus* infection in the skin. Furthermore, at 6 hrs after inoculation, *S. aureus*-infected skin lesions of mice deficient in TLR2, NOD2 or FPR1 had significant reductions in IL-1β protein (80, 62, and 91 percent decrease, respectively) and MPO activity (53, 58, and 47 percent decrease, respectively) compared with wt mice (Fig. 5C, D). Therefore, in addition to having higher *in vivo* bacterial burden, mice deficient in TLR2, NOD2 and FPR1 also had decreased IL-1β production and neutrophil recruitment during a *S. aureus* skin infection *in vivo*. The increased lesion sizes, higher bacterial burden and impaired IL-1β production in TLR2- and NOD2-deficient mice in response to *S. aureus* skin infection is consistent with previously published studies from our laboratory and others [3], [15].

### Mouse Neutrophils Directly Produce IL-1β in Response to *S. aureus*

**Figure 6:**
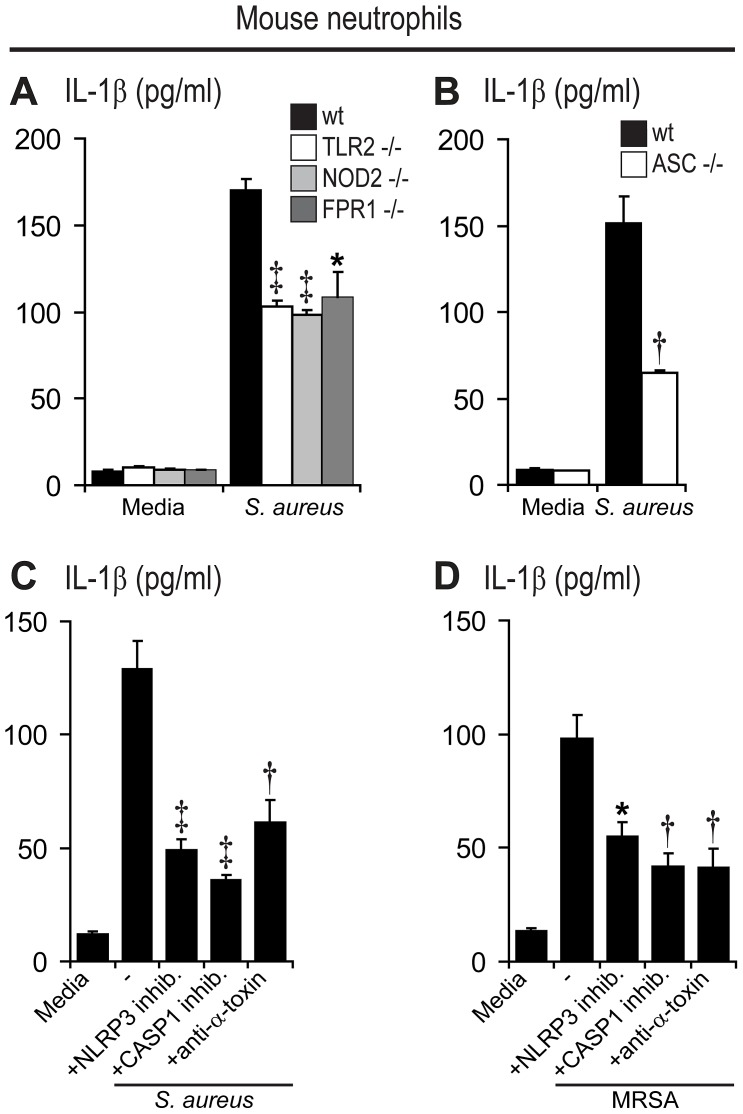
PRRs, α-toxin and the ASC/NLRP3 inflammasome contribute to *S. aureus*- and MRSA-induced IL-1β production by mouse neutrophils. Neutrophils from mouse bone marrow were infected with live *S. aureus* (SH1000) or MRSA (USA300 LAC strain) (MOI bacteria∶neutrophils of 5∶1) for a total culture time of 6 hrs and gentamicin was added at 60 min from the start of the infection to prevent bacterial overgrowth. IL-1β protein levels (mean ± SEM) were measured in culture supernatants by ELISA. (A, B) IL-1β protein levels in supernatants from *S. aureus*-infected neutrophils from TLR2^−/−^, NOD2^−/−^, FPR1^−/−^, ASC^−/−^ and wt mice. (C, D) IL-1β protein levels in supernatants from wt mouse neutrophils infected with *S. aureus* (C) or MRSA (D), in the absence and presence of an NLRP3-inhibitor (glibenclamide), a caspase-1 inhibitor (Z-YVAD-FMK) or anti-staphylococcal α-toxin antiserum. The respective vehicle controls (DMSO or normal rabbit IgG) for the inhibitors had IL-1β protein levels that did not differ than media alone (data not shown). Data are from 3–5 mice per group. *p<0.05; ^†^p<0.01, ^‡^p<0.001, TLR2^−/−^, NOD2^−/−^, FPR1^−/−^ or ASC^−/−^ mice versus wt mice (A, B) or the NLRP3-inhibitor, caspase-1 inhibitor or anti-staphylococcal α-toxin antiserum treatment versus media alone (C, D) (Student's *t*-test).

To determine whether IL-1β production by neutrophils occurred through direct or indirect mechanisms, neutrophils obtained from bone marrow of wt mice and mice deficient in TLR2, NOD2 or FPR1 (purity>99%) were infected with live *S. aureus in vitro* (Fig. 6). This *in vitro* infection involved incubating the neutrophils with live *S. aureus* or a community-acquired MRSA strain (USA300 LAC isolate) (at a multiplicity of infection [MOI] of bacteria to neutrophils of 5∶1) for a total of 6 hrs and gentamicin was added at 60 min from the start of the infection as previously described [16]. The levels of IL-1β protein produced in these cultures were measured using an ELISA that detects both pro-IL-1β and cleaved IL-1β. During this *in vitro* infection, we observed increased production of IL-1β protein as the MOI increased from 1∶1 to 5∶1 (Fig. S9A, B). The increased production of IL-1β was not due to increased cell death as there was no decrease in the viability of the neutrophils in the presence of *S. aureus* or MRSA compared with cultures without any bacterial infection (Fig. S9C, D). Furthermore, the lack of any decrease in viability of the neutrophils in the presence of *S. aureus* or MRSA suggest that the ELISA likely detected mostly cleaved IL-1β rather than pro-IL-1β released into the supernatants from dying cells. Using this *in vitro* infection, neutrophils from mice deficient in TLR2, NOD2 or FPR1 produced significantly less IL-1β protein (40, 43 and 37 percent decrease, respectively) in response to *S. aureus* (Fig. 6A), suggesting that activation of TLR2, NOD2 and FPR1 promoted neutrophil production of IL-1β. To provide further evidence that neutrophils and not other contaminating cells such as monocytes produced IL-1β in these cultures, purified neutrophils from pIL1-DsRed reporter mice were evaluated in this *in vitro* infection and 43% of the Ly6G^+^ neutrophils expressed IL-1β-DsRed whereas only 0.2% of other cell types (Ly6G^−^ cells) expressed IL-1β-DsRed (Fig. S10). Thus, neutrophils, and not other contaminating cells, were the predominant source of IL-1β in these cultures.

An important step in the production of active IL-1β is the enzymatic processing of pro-IL-1β into its active form. Typically, this cleavage is mediated by caspase-1, which is activated by an intracellular complex of proteins called the inflammasome [29]. However, under certain conditions, cleavage of pro-IL-1β into active IL-1β in neutrophils can be mediated by serine-proteases (such as proteinase 3) or neutrophil elastase rather than inflammasome/caspase-1 activation [30]–[32]. To determine whether IL-1β production by neutrophils in response to *S. aureus* involved inflammasome activation, we studied neutrophils from mice deficient in ASC, which is required for NLRP3 inflammasome assembly [33]. Neutrophils from wt or ASC-deficient mice were infected with live *S. aureus in vitro* (Fig. 6B). Neutrophils from ASC-deficient mice had a 58% decrease in IL-1β production in response to *S. aureus* compared with neutrophils from wt mice, indicating that the majority of IL-1β produced during the *S. aureus in vitro* infection was dependent on the inflammasome component ASC.

Previous studies in mouse and human monocyte/macrophage cultures demonstrated that processing of pro-IL-1β after exposure to *S. aureus* was dependent upon ASC/NLRP3 inflammasome and caspase-1 activation, which was induced by *S. aureus* α-toxin and other pore-forming hemolysins [17], [34]. Therefore, to evaluate whether a similar mechanism of inflammasome activation and IL-1β production was involved in mouse neutrophils, mouse neutrophils were infected with *S. aureus* or MRSA *in vitro* in the presence an inhibitor of the NLRP3 inflammasome (glibenclamide) [35], [36], a specific caspase-1 inhibitor (Z-YVAD-FMK) or neutralizing antibodies directed against *S. aureus* α-toxin [37] (Fig. 6C, D). In mouse neutrophils infected with *S. aureus*, the amount of IL-1β produced was decreased 62%, 73% and 53% by the NLRP3 inflammasome inhibitor, the caspase-1 inhibitor and the α-toxin neutralizing antibodies, respectively (Fig. 6C). Similarly, in mouse neutrophils infected with MRSA, the amount of IL-1β produced was decreased 44%, 57% and 58% by the NLRP3 inflammasome inhibitor, the caspase-1 inhibitor and the α-toxin neutralizing antibodies, respectively (Fig. 6D). Importantly, addition of inhibitors or neutralizing antibodies did not decrease the viability of neutrophils infected with *S. aureus* or MRSA (Fig. S11). To confirm that *S. aureus* or MRSA infection of mouse neutrophils resulted in the generation of cleaved IL-1β, immunoblotting was performed and cleaved IL-1β was only detected in cultures of *S. aureus*-or MRSA-infected neutrophils and not in uninfected neutrophil cultures (Fig. S12). In these mouse neutrophil cultures, there was no decrease in IL-1β produced in control wells containing DMSO (the vehicle for the NLRP3 inflammasome inhibitor and the caspase-1 inhibitor) or rabbit IgG (the control for the α-toxin neutralizing antibodies) compared with media alone (data not shown). Taken together, these data indicate that the majority of the IL-1β produced was dependent upon activation of caspase-1 via induction of the NLRP3/ASC inflammasome in an α-toxin-dependent mechanism. Furthermore, the dependence of IL-1β production on the NLRP3/ASC inflammasome and caspase-1 provides additional evidence that the IL-1β detected by the ELISA was mostly cleaved IL-1β rather than pro-IL-1β.

It should be noted that it was necessary to use methods that yielded purified cultures of mouse neutrophils for the *in vitro* infection experiments to minimize monocyte contamination. Mouse neutrophils were positively selected from bone marrow cells using anti-Ly6G magnetic bead separation. This method resulted in 99.1% purity of mouse neutrophils (Fig. S13). Although the positive selection with anti-Ly6G magnetic bead separation may have induced some activation of the mouse neutrophils, this degree of activation was unlikely to play a major role in production of IL-1β because there was minimal IL-1β production observed in cultures of uninfected neutrophils (Fig. 6A–D).

## Discussion

Neutrophil abscess formation is an essential component of innate immunity against many pathogens [1]. In this study, using gene expression analysis and advanced techniques of *in vivo* fluorescence imaging, we found that neutrophil recruitment during a *S. aureus* cutaneous infection is functionally and temporally linked to IL-1β/IL-1R activation. Based on our prior work [3], [11], we hypothesized that this association was a result of IL-1β production by hematopoietic cells such as macrophages or dendritic cells and possibly other cells that reside in the skin, such as keratinocytes or mast cells [20]–[23]. Surprisingly, we found that neutrophils were the most abundant source of IL-1β during infection. Neutrophil-derived IL-1β, in the absence of other cellular sources of IL-1β, was critical for host defense since adoptive transfer of IL-1β-expressing neutrophils was sufficient to restore the impaired neutrophil recruitment and abscess formation in *S. aureus*-infected IL-1β-deficient mice. In addition, mouse neutrophils produced IL-1β *in vitro* in response to live *S. aureus* in a mechanism involving the PRRs, TLR2, NOD2 and FPR1, and the ASC/NLRP3 inflammasome. Thus, neutrophil recruitment and IL-1β/IL-1R are functionally, temporally and spatially linked because neutrophils are the predominant source of IL-1β. These findings provide a new paradigm for abscess formation during an infection in which the inflammatory mediators produced by the epithelial, stromal and resident immune cells in the infected tissue may contribute to the recruitment of the very first neutrophils; however, this response is not sufficient for effective abscess formation. Rather, a feed-forward mechanism that involves early recruited neutrophils serving as a source of IL-1β is essential for amplifying and sustaining the neutrophilic response to promote optimal abscess formation and bacterial clearance.

Although a link between neutrophil recruitment and IL-1β during *S. aureus* infections was previously documented [7], [10], [11], our work defines the primary mechanism by which effective neutrophil abscess formation occurs. These findings provide an explanation for a number of puzzling observations in humans and mice and have important implications for neutrophil-derived IL-1β in potentially contributing to other immune responses during infection and inflammation. First, human pediatric patients with deficiency in TLR/IL-1R signaling molecules, MyD88 or IRAK-4, are predisposed to pyogenic bacterial infections, including *S. pneumoniae*, *S. aureus*, and *P. aeruginosa*, whereas other types of bacterial, fungal and viral infections are exceedingly rare [38], [39]. The reason for this has remained elusive, especially since these patients do not have impaired neutrophil number or function as seen in other conditions predisposed to pyogenic infections such as severe congenital neutropenia or chronic granulomatous disease [9]. Interestingly, during acute or invasive infections, patients with MyD88 or IRAK-4 deficiency develop neutropenia despite having pus in infected tissues [40]. Our findings suggest a pathway beginning with *S. aureus*-induced inflammation in the skin tissue that results in an initial early recruitment of neutrophils that produce IL-1β. The neutrophil-derived IL-1β is sufficient to amplify and sustain their recruitment that promotes neutrophilia and effective neutrophil abscess formation. Although patients with MyD88 or IRAK-4 deficiency may recruit neutrophils to the site of infection, they cannot respond to neutrophil-derived IL-1β to amplify the neutrophilic response, providing a potential explanation for their selective predisposition to pyogenic infections.

Second, previously published work has demonstrated that neutrophils express pattern recognition receptors, including TLR2 [41], [42], NOD2 [43] and FPRs [28]. Thus, we evaluated TLR2-, NOD2-, or FPR1-deficient mice in response to *S. aureus* skin infection and found that each of these mice had impaired IL-1β production (Fig. 5C) and neutrophil recruitment after *S. aureus* skin infection (Fig. 5D). Based on our *in vitro* infection experiments with neutrophils from TLR2-, NOD2-, or FPR1-deficient mice, there was decreased IL-1β production compared with neutrophils from wt mice (Fig. 6A), suggesting these each of these PRRs on neutrophils directly contribute to the production of IL-1β in response to *S. aureus*. Since TLR2, NOD2 and FPR1 are activated by different bacterial components, they likely provide overlapping and redundant functions to ensure adequate neutrophil IL-1β production and a deficiency in any one of these PRRs would not have a major impact on host defense. However, TLR2, NOD2 and FPR1 have also been shown to also be involved in other neutrophil functions such as chemotaxis, phagocytosis and oxidative burst [14], [44]–[47]. Thus, the impaired immune response against *S. aureus* in TLR2-, NOD2-, or FPR1-deficient mice *in vivo* may not be solely due to decreased IL-1β production but is likely dependent upon the lack of other functional activities of these PRRs on neutrophils as well as on other cell types in the infected skin in these knockout mice. To determine if TLR2 functioned predominantly on neutrophils or other cell types *in vivo*, we performed an additional experiment in which wt neutrophils were adoptively transferred into TLR2-deficient mice (Fig. S14). This adoptive transfer of wt neutrophils did not rescue the immune impairment in TLR2-deficient mice as observed with the adoptive transfer of wt neutrophils into IL-1β-deficient mice (Fig. 4). Thus, TLR2 activation is needed on other cells to invoke protective mechanisms *in vivo*.

Third, our previous work found that production of IL-1β was likely from bone marrow-derived hematopoietic cells because neutrophil recruitment, host-defense and IL-1β production at the site of infection was restored in IL-1β-deficient mice reconstituted with bone marrow from wt mice but not IL-1β-deficient mice [11]. Here, we found that adoptively transferred wt neutrophils could rescue the immune impairments in IL-1β-deficient mice, indicating neutrophils are the predominant hematopoietic cellular source of IL-1β that was sufficient for effective neutrophil recruitment and abscess formation. These data further argue against an important role for IL-1β produced by non-hematopoietic cells during the *S. aureus* skin infection. Although keratinocytes produced IL-1β during the infection as detected by immunohistochemistry (Fig. S15), this amount of IL-1β was not able to promote effective neutrophil recruitment in the absence of IL-1β-expressing hematopoietic cells because wt mice reconstituted with bone marrow from IL-1β-deficient mice show the same impaired neutrophil recruitment response as normal non-irradiated/non-reconstituted IL-1β-deficient mice [11].

Fourth, IL-1 has been shown to play a role in neutrophil recruitment during sterile inflammation. In a mouse model of intraperitoneal injection of necrotic lymphoma cells or acetaminophen-induced liver injury, neutrophil recruitment to the peritoneal cavity or liver was mediated by IL-1α alone or both IL-1α and IL-1β, respectively [48]. Interestingly, in a mouse model of autoimmune inflammatory arthritis, neutrophil recruitment to the inflamed joints was dependent on leukotriene B_4_ (LTB_4_) [49]. However, exogenous IL-1β injected into the joints or adoptive transfer of wt neutrophils could restore neutrophil recruitment and arthritis in LTB_4_-deficient mice, demonstrating that IL-1β-producing neutrophils amplified neutrophil recruitment and arthritis [49], which was similar to what we observed during a *S. aureus* skin infection. Thus, although IL-1β-producing neutrophils are sufficient for neutrophil recruitment during a *S. aureus* skin infection, neutrophil recruitment during sterile inflammation is mediated by IL-1α, IL-1β or both IL-1α and IL-1β, depending on the anatomical site and the type of inflammation.

Neutrophil-derived IL-1β may also promote other immune responses at the site of infection. Similar to our findings, a previous report found that NOD2-deficient mice had impaired production of IL-1β during a *S. aureus* skin infection [15]. This report also found that NOD2-induced IL-1β contributed to production of IL-6, which enhanced neutrophil killing of *S. aureus* [15]. In our previous work, we found that IL-1R-mediated neutrophil recruitment (through production of the neutrophil-attracting chemokines KC and MIP2) was dependent upon IL-1R-signaling by resident skin cells rather than bone marrow-derived recruited cells [3]. These findings were based upon data using bone marrow chimeric mice in which the impaired host defense and neutrophil recruitment in IL-1R-deficient mice could not be restored in IL-1R-deficient mice reconstituted with bone marrow from wt mice [3]. In contrast, wt mice reconstituted with bone marrow from IL-1R-deficient had no immune impairment. Additionally, as mentioned above, IL-1β production during the *S. aureus* skin infection was found to be dependent on bone marrow-derived cells rather than resident skin cells [11]. Combining these previous studies with the present findings, a host defense pathway has been discovered whereby neutrophils represent a source of IL-1β, which subsequently activates IL-1R expressed on non-bone marrow-derived resident skin cells to promote effective neutrophil recruitment in host defense during a *S. aureus* skin infection. Furthermore, we had previously demonstrated that IL-1R activation was required for inducing IL-17A/F production by γδ T cells in infected mouse skin at early time points after *S. aureus* infection [19]. In this context, IL-17A/F promoted enhanced neutrophil recruitment via induction of neutrophil-attracting chemokines and granulopoiesis factors. Since IL-1β has also been shown to be important in the generation of Th17 cells [50], [51], future studies will be required to determine if neutrophil-derived IL-1β contributes to the IL-6 responses as well as the development of Th17 cells and other IL-17-producing cells following a cutaneous *S. aureus* infection.

It should also be noted that since IL-1β- and IL-1R-deficient mice ultimately clear these infections, compensatory mechanisms exist that eventually promote bacterial clearance. Similar compensatory mechanisms also may play a role in TLR2-, NOD2- and FPR1-deficient mice as these mice also eventually clear the infection and the cellular composition of 7/4^+^ neutrophils and MOMA2^+^ monocytes/macrophages on day 10 after infection in TLR2-, NOD2-, FPR1-deficient mice was similar to the cellular composition in wt mice whereas IL-1β-deficient mice had a paucity of 7/4^+^ neutrophils at this time point (Fig. S16). These compensatory responses may include activation of other MyD88-dependent receptors such as TLRs, IL-18 or IL-33 because we found that MyD88-deficient mice have a more severe impairment in neutrophil recruitment than IL-1β- or IL-1R-deficient mice [3], [11]. In addition, IL-17 has also been shown to be critical in promoting neutrophil recruitment and antimicrobial responses against *S. aureus* in various mouse models of infection (cutaneous infection, systemic infections, pneumonia and brain abscesses [52]–[56]) as well as in humans with hyper-IgE syndrome or with a deficiency in IL-17F or IL-17RA [50], [57]–[60]. Although Th17 development is severely impaired in IL-1R-deficient mice *in vivo* [61], [62] and γδ T cell production of IL-17 is enhanced in the presence of IL-1β [19], [63], the numbers and activity of Th17 and γδ T cells may increase during the course of the *S. aureus* skin infection and compensate for absence of IL-1β activity. Consistent with this possibility, humans with deficiency in the IL-1R downstream signaling molecules MyD88 or IRAK-4 do not exhibit impaired development of IL-17-producing cells [64].

The use of *in vivo* imaging in this study to quantify the bacterial burden, neutrophil recruitment and IL-1β production provides an approximation of these endpoints and there are advantages and limitations that should be considered when interpreting this data. First, *in vivo* bioluminescence imaging provides only a close estimate of *in vivo* bacterial burden as several factors such as body temperature, metabolic activity of the bacteria *in vivo* [65], [66] and the presence of reactive oxygen mediators produced by neutrophils at the site of infection that could potentially react with the bacterial luciferase as seen with GFP-labeled bacteria [67], [68]. However, despite these potential confounding factors, we previously demonstrated that *in vivo* bioluminescent signals directly correlate with *ex vivo* CFUs harvested at different time points from the *S. aureus*-infected skin lesions [3], [19], [69]. Thus, the bioluminescent signals and actual bacterial burden is not a perfect correlation; however, it is a noninvasive method that approximates the bacterial burden *in vivo* that does not require euthanasia of numerous animals at every time point to obtain this information. Second, regarding the use of the LysEGFP mice, lysozyme M is expressed in myeloid cells (including neutrophils and monocytes/macrophages) and the lysozyme M promoter driven EGFP expression is not specific for neutrophils [25]. However, neutrophils from LysEGFP mice have been shown to have much brighter EGFP fluorescence intensity than monocytes or macrophages [70], [71] and we found that F4/80^+^ macrophages constituted less than 10% of the EGFP-expressing cells during the first 5 days after skin wounding of LysEGFP mice [72], indicating that neutrophils may contribute to more than 90% of the EGFP signals. In addition, the decreasing EGFP signals from days 1 to 10 correlated with the decreasing numbers of 7/4^+^ neutrophils and not the increasing numbers of MOMA2^+^ monocytes/macrophages at these time points as detected by immunohistochemistry (Fig. 3B and S16). Furthermore, EGFP was expressed within the cytoplasm and intracellular vesicles of LysEGFP neutrophils and the intensity of EGFP fluorescent signals was not substantially decreased in culture after neutrophil degranulation induced in response to fMLF or PMA (Fig. S17). Thus, the EGFP fluorescent signals more closely approximate of the numbers of neutrophils within the infected skin during the course of infection. With respect to using pIL1-DsRed transgenic mice, there are slightly different kinetics between DsRed fluorescence and IL-1β protein expression measured by ELISA, since DsRed fluorescence in extracts of inflamed skin and in cell culture was induced 6–12 hrs slower and persisted ∼24 hrs longer than IL-1β protein levels [21]. However, the difference in kinetics of DsRed fluorescence signals were less than 24 hrs and thus would be unlikely to impact the approximation of IL-1β production *in vivo*, since we began our measurements 1 day after infection and the infection takes over 14 days to resolve. Furthermore, there was substantial DsRed fluorescence signal on day 1 during our *in vivo S. aureus* skin infection (Figs. 2 and 3) and at this time point we previously found that IL-1β was detected from the infected skin by ELISA and that both pro-IL-1β and cleaved IL-1β were detected by immunoblotting [11], [17].

In the present study, we found that processing of pro-IL-1β into active IL-1β by mouse neutrophils was largely dependent upon ASC/NLRP3 inflammasome activation *in vitro*. We further demonstrated that IL-1β production was dependent upon the activity of α-toxin. These data are consistent with previous work in human or mouse monocyte/macrophage cultures demonstrating that processing of pro-IL-1β during *S. aureus* cutaneous infections *in vivo* was dependent upon ASC/NLRP3 inflammasome and caspase-1 activation, which was induced by *S. aureus* pore-forming toxins (i.e. α-, β- and γ-hemolysins) or digestion of peptidoglycan mediated by lysozyme [17], [18], [34]. Since there was some IL-1β measured in cultures of neutrophils from ASC-deficient mice as well as in cultures of wt neutrophils in the presence of the NLRP3 or caspase-1 inhibitor, the remainder of IL-1β may have been produced through an inflammasome-independent pathway mediated by serine-proteases (such as proteinase 3) or neutrophil elastase [30]–[32]. In addition, the residual IL-1β detected in these cultures may also reflect transcription of pro-IL-1β since the ELISA detects both pro-IL-1β and cleaved IL-1β.

In the adoptive transfer experiments, although the expression of IL-1β protein at the site of infection was extremely low compared with the levels observed in wt mice, scattered IL-1β-producing cells were detected by immunohistochemistry in IL-1β-deficient mice adoptively transferred with wt neutrophils (Fig. S8). While it is tempting to speculate that these adoptively transferred IL-1β-producing neutrophils directly rescued the neutrophil recruitment response at the site of infection, it is also possible the low levels of IL-1β acted indirectly and/or through another anatomical site such as the blood [73]. Future studies will be necessary to further dissect the mechanism by which neutrophil-derived IL-1β contributes to host defense during a *S. aureus* skin infection. Nevertheless, we show that IL-1β-producing adoptively transferred neutrophils were sufficient to rescue the impaired immunity in IL-1β-deficient mice. These findings provide evidence that neutrophil-derived IL-1β can promote effective neutrophil abscess formation and host defense against a cutaneous *S. aureus* infection.

IL-1β production in response to live *S. aureus* cultured with mouse bone marrow-derived macrophages (BMDMs), mouse peritoneal macrophages or human monocytes has previously been described [16]–[18], [74]. These studies used different *S. aureus* strains, MOIs and culture conditions and the amount of IL-1β produced was generally 15- to 100-fold greater than the levels we observed with our *in vitro* infection of mouse neutrophils with *S. aureus* or MRSA. To evaluate whether IL-1β produced by the few contaminating monocytes/macrophages played any role in the adoptive transfer experiments, monocytes were specifically depleted from the adoptively transferred cells and the lack of monocytes had no impact on the ability of the adoptively transferred wt neutrophils to rescue the immune impairment in IL-1β-deficient mice. Furthermore, our *in vitro* infection experiments used highly purified mouse neutrophils separated with anti-Ly6G MACS magnetic beads (99.1% pure with only 0.1% monocytes) to ensure that we were evaluating neutrophil specific production of IL-1β. To provide additional evidence that neutrophils and not other contaminating monocytes produced IL-1β in these cultures, purified neutrophils from pIL1-DsRed reporter mice were evaluated and 43% of the neutrophils expressed IL-1β-DsRed whereas only 0.2% of other cell types expressed IL-1β-DsRed. Taken together, these data indicate that neutrophils were the predominant source of IL-1β for both the adoptive transfer experiments and the *in vitro* infection experiments. Finally, a recent study evaluated sorted mouse bone marrow cells to determine which cell type produced the majority of IL-1β in response to LPS in the presence of the known inflammasome activators ATP or nigericin [75]. They found neutrophils were the predominant source of IL-1β as they produced almost 3-fold more IL-1β than F4/80^+^ macrophages. They further demonstrated that human neutrophils were responsible for half of all IL-1β secreted by human PBMCs. Lastly, they showed that IL-1β production by mouse and human neutrophils involved activation of inflammasome via NLRP3/ASC/caspase-1 axis. These data are consistent with our findings that neutrophils provide a major source of IL-1β during a *S. aureus* skin infection that is produced in an NLRP3/ASC/caspase-1-dependent manner

In summary, we have identified that neutrophil-derived IL-1β is essential for amplifying the neutrophilic response to promote abscess formation and clearance of a *S. aureus* skin infection. From a clinical point of view, these findings provide the basis for targeting IL-1β production by neutrophils to improve immunity against pyogenic infections, especially in patients with impaired neutrophilic responses.

## Materials and Methods

### Ethics Statement

All animals were handled in strict accordance with good animal practice as defined in the federal regulations as set forth in the Animal Welfare Act (AWA), the 1996 Guide for the Care and Use of Laboratory Animals, PHS Policy for the Humane Care and Use of Laboratory Animals, as well as UCLA's policies and procedures as set forth in the UCLA Animal Care and Use Training Manual, and all animal work was approved by the UCLA Chancellor's Animal Research Committee (ARC#: 2008-099).

### *Staphylococcus aureus* Strains

The bioluminescent *S. aureus* SH1000 strain ALC2906, which possesses the shuttle plasmid pSK236 with the penicillin-binding protein 2 (*pbp2*) promoter fused to the modified *luxABCDE* reporter cassette from *Photorhabdus luminescens*, was used as a representative *S. aureus* strain [3]. This strain emits bioluminescence signals from live, actively metabolizing bacteria in all stages of the *S. aureus* life cycle. In some experiments, a community-acquired MRSA strain was used (USA300 LAC isolate [76]), which was kindly provided by Frank DeLeo (National Institute of Allergy and Infectious Diseases, Rocky Mountain Laboratories in Hamilton, MT).

### Preparation of *S. aureus* for Skin Inoculation

SH1000 cultures were grown in the presence of chloramphenicol (10 µg/ml; Sigma-Aldrich, St. Louis, MO). *S. aureus* or MRSA was streaked onto tryptic soy agar (tryptic soy broth [TSB] plus 1.5% bacto agar; BD Biosciences, Sparks, MD) and single colonies were placed into TSB and grown overnight at 37°C in a shaking incubator. Mid-logarithmic phase bacteria were obtained after a 2 hr subculture of a 1∶50 dilution of the overnight culture. Bacterial cells were pelleted, resuspended, and washed 3 times in PBS. Bacterial concentrations were estimated by measuring the absorbance at 600 nm (A_600_) (Biomate 3; Thermo Scientific, Waltham, MA). In some experiments, bacteria was heat-killed (65°C for 30 minutes) prior to infection. CFUs were verified by plating dilutions of the inoculum overnight.

### Mice

Male mice on a C57BL/6 genetic background were used in all experiments. pIL1-DsRed-reporter mice [21], LysEGFP mice [25], FPR1-deficient mice [77], IL-1β-deficient mice [78] and ASC-deficient mice [33] were generated as previously described. IL-1R1-deficient mice (B6.129S7-Il1r1^tm1Imx^/J), TLR2-deficient mice (B6.129-TLR2^tm1Kir^/J) and NOD2-deficient mice (B6.129S1-Nod2^tm1Flv^/J) and wt C57BL/6 mice were obtained from Jackson Laboratories (Bar Harbor, ME). All mouse colonies were maintained in autoclaved cages under specific-pathogen free conditions.

### Mouse Model of Cutaneous *S. aureus* Infection

The mice were shaved on the back and inoculated intradermally with mid-logarithmic growth phase *S. aureus* (2×10^6^ CFUs) in 100 µl of sterile saline using a 27-gauge insulin syringe as previously described [3]. Measurements of total lesion size (cm^2^) were made by analyzing digital photographs of mice using the software program Image J (http://rsbweb.nih.gov/ij/).

### Gene Microarray Analysis: Gene Induction, Functional Groups and Network Analysis

Skin punch biopsy (8-mm) specimens from uninfected or lesional skin were taken at 4 hrs after *S. aureus* intradermal inoculation from wt and IL-1R-deficient mice and homogenized (Bio-Gen Pro200; Pro Scientific, Oxford, CT). RNA was isolated using TRIzol reagent (Invitrogen, Grand Island, NY) and purified using the RNeasy Mini kit (Qiagen, Valencia, CA). The UCLA Microarray Core performed probe synthesis and hybridization to the GeneChip Mouse Genome 430 2.0 Array (Affymetrix, Maumee, OH) according to the manufacturer's protocol. Image files were processed using the invariant set method for probe selection during normalization and the model-based expression method of pooling information across arrays using dCHIP (DNA-Chip Analyzer) gene expression software (www.dchip.org) [79]. Genes were considered upregulated in *S. aureus*-infected skin at 4 hrs compared with uninfected skin according to the criteria: fold-change >1.5, p-value<0.05. Functional group and network analysis was performed using Ingenuity Pathway Analysis software (version 6.0; Ingenuity Systems, Redwood City, CA) as previously described [80]. The raw gene expression data for this study are available through the Gene Expression Omnibus database (http://www.ncbi.nlm.nih.gov/geo/) under accession number GSE36826.

### RNA Extraction and mRNA Quantification

Total RNA from homogenized (Pro200 Series homogenizer [Pro Scientific]) 8-mm skin biopsy specimens taken at 4 hrs from skin inoculated with live or heat-killed *S. aureus* and uninfected skin was extracted by the use of TRIzol reagent (Invitrogen), followed by DNase treatment (Invitrogen) according to the manufacturer's recommendations. Real-time quantitative real-time PCR (Q-PCR) reactions were performed as previously described [19]. TaqMan Gene Expression Assays primers and probes sets for a subset of genes of interest in the Cell Movement of Neutrophils sub-group, including *IL-6*, *G-CSF*, *MIP-2β*, *MIP-2α*, *IL-1β*, *GM-CSF*, *CXCR2*, *G-CSF-R*, *TLR2* and *LTB4R* and the normalizer *GAPDH* were purchased from Applied Biosystems (Foster City, CA). The relative quantities of mRNA per sample were determined using the ΔΔC(T) formula as previously described [3]

### Quantification of *In Vivo S. aureus*: *In Vivo* Bioluminescence Imaging

Mice were anesthetized via inhalation of isoflurane and *in vivo* bioluminescence imaging was performed using the IVIS Lumina II imaging system (Caliper Life Sciences, a PerkinElmer Company, Alameda, CA) as previously described [3]. Data are presented on color scale overlaid on a grayscale photograph of mice and quantified as total flux (photons/s) within a circular region of interest using Living Image software (Caliper).

### Quantification of IL-1β Production and Neutrophil Infiltration: *In Vivo Fluorescence* Imaging

pIL1-DsRed mice and LysEGFP mice were anesthetized with inhalation isoflurane and *in vivo* fluorescence imaging was performed (sequentially after *in vivo* bioluminescence imaging) using the IVIS Lumina II imaging system (Caliper). DsRed fluorescence was measured using: excitation (535 nm), emission (575–650 nm) and exposure time (0.5 s). EGFP fluorescence was measured using: excitation (465 nm), emission (515–575 nm) and exposure time (0.5 s). Data are presented on color scale overlaid on a grayscale photograph of mice and quantified as total radiant efficiency ([photons/s]/[µW/cm^2^]) within a circular region of interest using Living Image software (Caliper).

### Tissue Embedding and Staining

For histological analysis, lesional 8-mm punch biopsy skin specimens were embedded in Tissue-Tek OTC compound (Sakura Finetek) and cut into 4 µm sections by the UCLA Tissue Procurement and Histology Core Laboratory, according to guidelines for clinical samples.

### Immunofluorescence and Confocal Laser Microscopy

Frozen sections were fixed in acetone, air-dried, and rehydrated in PBS. Sections were permeabilized with 0.1% saponin in PBS and non-specific binding was blocked with 2% goat serum (Invitrogen) and mouse IgG2a (10 µg/ml; clone UPC 10, Sigma-Aldrich) in PBS. Sections were subsequently labeled with primary antibodies specific for DsRed (rabbit anti-DsRed antibody; Clontech, Mountain View, CA) in combination with mAbs specific for neutrophils (anti-7/4 [Ly-6B.2]; 5 µg/ml; AbD Serotec, Raleigh, NC), monocytes/macrophages (anti-MOMA2; 5 µg/ml; AbD Serotec), antigen presenting cells (anti-MHC II; 5 µg/ml; clone 2G9; BD Biosciences) or total leukocytes (anti-CD45; 5 µg/ml; clone 30-F11; BD Biosciences) or appropriate isotype controls. Secondary antibodies included goat anti-rabbit IgG-Alexa 568 and goat anti-rat IgG-Alexa 488 (Invitrogen, Carlsbad, CA). All specimens were imaged on a Leica SP2-1P FCS Confocal Microscope (Leica Microsystems, Heidelberg, Germany) as previously described [19]. Representative images of isotype controls are shown (Fig. S4B, D). Quantification of co-localization was performed using the Manders' coefficient for a value range of 0 to 1 in which 0 = no pixels co-localize and 1 = all pixels co-localize using Definiens Tissue Studio software (Definiens, Parsippany, NJ). The Manders' coefficient was determined from 4 different mice per group after averaging 2–3 fields of view per specimen.

### Immunoperoxidase Labeling

Detection of 7/4^+^ or MOMA2^+^ cells on frozen sections of lesional skin was performed with the anti-7/4 mAb or the anti-MOMA2 mAb as described above, followed by the biotinylated goat anti-rat IgG polyclonal antibody (5 µg/ml; Vector Labs, Burlingame, CA) or corresponding isotype control antibodies. To detect IL-1β protein expression, a biotinylated anti-mouse IL-1β (20 µg/ml: clone 1400.24.17; Thermo Scientific) or corresponding isotype control mAb was employed. All procedures were performed using the immunoperoxidase method as previously described [11].

### Myeloperoxidase (MPO) Assay

MPO activity in lesional skin specimens was determined using an established MPO activity assay. Briefly, 8-mm punch biopsies were weighed and homogenized (Bio-Gen Pro200; Pro Scientific) in a buffer containing potassium phosphate (50 mM, pH 6.0) and hexadecyltrimethylammonium bromide (0.5%; Sigma-Aldrich). To measure MPO levels, 140 µl assay buffer, containing *o*-Dianisidine dihydrochloride (0.168 mg/ml; Sigma-Aldrich) and hydrogen peroxide (0.05%; Sigma-Aldrich), was added to 10 µl of homogenized supernatant and the change in absorbance (A_490_) was determined at 40 s intervals for 2 min using the Synergy 2 microplate reader (BioTek, Winooski, VT). Purified MPO (Sigma-Aldrich) was used to generate a standard curve and data are presented as MPO activity (U/mg tissue weight).

### Flow Cytometry

For mouse neutrophils, the expression of the neutrophil-specific marker Ly6G (FITC-conjugated rat anti-Ly6G mAb; 5 µg/ml; clone 1A8, IgG1; BD Pharmingen) and the monocyte-specific marker CD115 (the M-CSF receptor) (PE-conjugated rat anti-CD115 mAb, 2 µg/ml; clone AFS98; eBioscience), CD11b (APC-conjugated rat anti-CD11b mAb; 2 µg/ml; clone M1/70; BD Pharmingen) and corresponding fluorescently-conjugated isotype control mAbs were used. Using these antibodies, Ly6G^+^ CD115^−^ or Ly6G^+^ CD11b^high^ represented mouse neutrophils and Ly6G^−^ CD115^+^ or Ly6G^−^ CD11b^low^ represented mouse monocytes as previously described [81]–[83]. In some experiments, purified neutrophils from pIL1-DsRed reporter mice were used. pIL1-DsRed neutrophils were co-labeled with the anti-Ly6G mAb and prepared for flow cytometry as described above.

### Mouse Neutrophil Isolation for Adoptive Transfer Experiments

For adoptive transfer experiments, neutrophils were obtained from the bone marrow of IL-1β-deficient or wt mice using Percoll density gradient centrifugation. Briefly, marrow cavities of the tibias and femurs of 8-week old mice were flushed with complete RPMI 1640 containing 10% FBS. After hypotonic lysis of red blood cells, mature neutrophils were isolated by centrifugation for 30 min at 10°C and 1600 g over a discontinuous Percoll gradient consisting of 50% (vol/vol), 55% (vol/vol), 62% (vol/vol) and 81% (vol/vol) Percoll (Sigma-Aldrich, St. Louis, MO) in PBS.

The purity of the adoptively transferred cells was determined by flow cytometry using the neutrophil specific marker Ly6G (anti-Ly6G mAb, clone 1A8) and the monocyte-specific marker (anti-CD115 mAb, the M-CSF receptor). These markers have been shown to distinguish between mouse neutrophils and monocytes by flow cytometry [81], [82]. The adoptively transferred cells were 90% neutrophils (Ly6G^+^ CD115^−^ cells), 0.26% monocytes (Ly6G^−^ CD115^+^ cells) and 9.1% Ly6G^−^ CD115^−^ cells, which likely represented other granulocytes (eosinophils or basophils) or residual red blood cells not lysed with the lysis buffer (Fig. S7A). After washing extensively in saline, 5×10^6^ adoptively transferred neutrophils in 100 µl of sterile saline were injected intravenously into IL-1β-deficient mice two hrs prior to intradermal inoculation with *S. aureus*. In some experiments, neutrophils or monocytes were specifically depleted prior to adoptive transfer using either an anti-Ly6G or an anti-CD115 MicroBead Kit and MACS magnetic bead separation (61.4% and 76.9 percent depletion efficiency, respectively) according to the manufacturer's protocols (Miltenyi Biotec, Inc., Auburn, CA) (Fig. S7B, C). In another set of adoptive transfer experiments, neutrophils were obtained from the bone marrow of TLR2-deficient or wt mice and adoptively transferred into TLR2-deficient recipient mice and the mice were infected with *S. aureus* according to the same procedures as described above (Fig. S14).

### Mouse Neutrophil Isolation for the *In Vitro S. aureus* or MRSA Infection

For all *in vitro* cultures with mouse neutrophils, neutrophils were obtained from the bone marrow of TLR2-, NOD2, FPR1- and ASC-deficient mice, pIL1-DsRed reporter mice or wt mice by anti-Ly6G MACs magnetic bead separation according to the manufacturer's protocols (Miltenyi Biotec, Inc.). Purity of the mouse neutrophils was determined by flow cytometry (see above) and these cultures contained 99.1% Ly6G^+^ CD11b^high^ neutrophils. There were very few Ly6G^−^ CD11b^+^ monocytes (0.1%) and the remaining cells (0.6%) were Ly6G^−^ CD11b^−^ cells (Fig. S13).

### Stimulation of Mouse Neutrophils with Live *S. aureus* or MRSA in Culture

Murine neutrophils (from TLR2-, NOD2-, FPR1-, ASC-deficient or wt mice) were cultured in RPMI 1640 complete media supplemented with 10% heat-inactivated FBS at a density of 1×10^5^ cells per 200 µl/well in a 96-well plate. These neutrophil cultures were infected with live *S. aureus* (SH1000 strain) or MRSA (USA300 LAC isolate) at a multiplicity of infection (MOI) of bacteria to neutrophils of 5∶1, 2∶1 or 1∶1 at 37°C and 5% CO_2_ in a humidified incubator for 6 hrs. Gentamicin (20 µg/ml) was added to the cultures at 60 minutes after infection according to previous methods to study inflammasome activation in response to live *S. aureus in vitro* [16]. Using these culture conditions, the MOI of 5∶1 for *S. aureus* or MRSA resulted in the highest production of IL-1β compared with MOI of 2∶1 or 1∶1 (Fig. S9A, B). The levels of IL-1β in wt mouse neutrophils did not differ more than 15% between experiments. There was also no decrease in neutrophil viability in any of the cultures with the different MOI of *S. aureus* or MRSA compared with neutrophils cultured in the absence of any bacteria (Fig. S9C, D). Therefore, the MOI of 5∶1 was used in all *in vitro* culture experiments. In some experiments, specific inhibitors were also added to the culture at the same time as *S. aureus* or MRSA. These include, the NLRP3-inhibitor, glibenclamide (100 µM; Imgenex) [35], [36], the caspase-1 inhibitor Z-YVAD-FMK (20 µM; Millipore), anti-staphylococcal α-toxin antiserum (1% vol/vol; Sigma-Aldrich) [37], or respective vehicle controls (DMSO or normal rabbit IgG).

### Cell Viability Assay

After *in vitro* infection of neutrophils with *S. aureus* or MRSA, cell viability was determined using the CellTiter 96 AQ_ueous_ One Solution Cell Viability Assay (Promega Corporation, Madison, WI) according to the manufacturer's instructions.

### Enzyme-Linked Immunosorbent Assays for IL-1β in Mouse Skin and Culture Supernatants

Protein levels of IL-1β from lesional mouse skin were obtained from tissue homogenates (Pro200 Series homogenizer [Pro Scientific]) of 8-mm skin punch biopsy specimens performed at 4 and 24 hrs after *S. aureus* skin inoculation using a commercially-available ELISA kit (R&D Systems). Levels of mouse IL-1β protein in culture supernatants were determined by using a commercially available ELISA kit (R&D Systems, Minneapolis, MN) according to the manufacturer's instructions.

### Immunoblotting for Detection of Pro-IL-1β and Cleaved IL-1β

For detection of pro-IL-1β (35 kDa) and cleaved IL-1β (17 kDa) by immunoblotting, purified mouse neutrophils from wt mice were cultured in RPMI 1640 media supplemented with 10% heat-inactivated FBS at a density of 1×10^6^ cells per 500 µl/well in a 24-well plate. These neutrophil cultures were infected with live *S. aureus* (SH1000 strain) or MRSA (USA300 LAC isolate) at an MOI of bacteria to neutrophils of 5∶1 at 37°C and 5% CO_2_ in a humidified incubator for 6 hrs and gentamicin (20 µg/ml) was added to the cultures at 60 minutes after infection. Following incubation, cells were lysed using the M-PER Mammalian Protein Extraction Reagent (Thermo-Fisher) supplemented with a Protease Inhibitor Cocktail (Sigma-Aldrich). Cell lysates were diluted in SDS-PAGE sample buffer, boiled for 5 min, and proteins were separated by SDS-PAGE. Pro-IL-1β and cleaved IL-1β protein were detected by immunoblotting using a goat anti-mouse IL-1β polyclonal antibody (1∶2000 dilution; catalog #: AF-401-NA; R&D Systems, Minneapolis, MN) followed by HRP-conjugated chicken anti-goat-HRP (1∶1000 dilution; catalog #: HAF019; R&D Systems).

### *In vitro* Functional Assays for Neutrophil Function (Phagocytosis, Degranulation, Oxidative Burst and Bacterial Killing)

All assays were performed using anti-Ly6G magnetic bead enriched neutrophils obtained from bone marrow cells of wt or IL-1β-deficient mice as described above. Phagocytosis was measured using pHrodo *S. aureus* BioParticles (Invitrogen), according to the manufacturer's instructions. Briefly, 1×10^5^ neutrophils were incubated with fluorophore-conjugated *S. aureus* bioparticles at 37°C for 1 hr. Cells were then stained with FITC-conjugated anti-Ly6G and pHrodo-positive neutrophils were quantified by flow cytometry. For neutrophil degranulation, 1×10^5^ neutrophils were stimulated for 30 min. at 37°C with 1 µM fMLF. Lactoferrin release was quantified from the supernatant using a mouse Lactoferrin ELISA kit (Biotang, Inc., Waltham, MA). Release of neutrophil reactive oxygen species was measured using the Phagoburst kit (Orpegen Pharma, Heidelberg, Germany) according to the manufacturer's instructions. Briefly, 5×10^5^ neutrophils were treated with 1 µM fMLF for 10 min at 37°C. The generation of reactive oxygen species was measured by flow cytometry by gating on 10,000 neutrophil events and determining the proportion of these cells positive for the conversion of the substrate dihydrorhodamine-123 to fluorescent rhodamine-123. Finally, neutrophil killing assays were performed by opsonizing *S. aureus* with 10% serum from C57BL/6 wt mice and adding the opsonized bacteria to purified neutrophils at a 1∶1 ratio (2×10^5^ neutrophils∶2×10^5^ CFU bacteria) for 45 min at 37°C. After incubation, neutrophils were diluted in H_2_O (pH 11) to lyse the neutrophils and serial dilutions were plated on TSB agar plates to enumerate viable bacterial CFU. As negative a control, bacteria were also incubated in media without neutrophils.

### Visualization of EGFP in Neutrophils from LysEGFP Mice and LysEGFP Neutrophil Degranulation Assays

Neutrophils were obtained from the bone marrow of LysEGFP mice using Percoll density gradient centrifugation. Neutrophils were washed once and resuspended in 1 ml of RPMI 1640 (Gibco) supplemented with 5 µg bisBenzinamide Hoescht 33342 trihydrochloride, a nuclear counterstain, (Sigma-Aldrich) for 30 minutes at room temperature. Neutrophils were subsequently attached onto glass slides using a Shandon Cytospin IV (Thermo Scientific) and imaged using an Olympus ×61 fluorescence microscope. To determine whether EGFP fluorescent signals were altered after neutrophil degranulation, neutrophils from LysEGFP mice were left unstimulated or stimulated with 1 µM fMLF or 100 ng/ml PMA (both from Sigma-Aldrich) for 15 minutes at 37°C. Cells were labeled with a biotinylated anti-mouse Ly6G (University of California San Francisco Monoclonal Antibody Core) with streptavidin-PE (Caltag) and anti-mouse PE-Cy7 CD11b (Biolegend) and analyzed on a Beckman Coulter FC500 flow cytometer.

### Statistical Analyses

Data were compared using Student's t test (2-tailed). All data are expressed as mean ± SEM (standard error of the mean) where indicated. Values of *p<0.05, †p<0.01, and ‡p<0.001 were considered statistically significant.

## Supporting Information

Figure S1 **Differentially expressed genes.** Genes differentially expressed between 0 and 4 hrs post-infection were identified according to the following criteria: fold change >1.5 and p-value<0.05. The heatmap was generated using the R statistical package (www.r-project.org).
(TIF)

Figure S2 **Comparison of gene expression between inoculation of live and heat-killed** ***S. aureus*** **in the skin of wt versus IL-1R-deficient mice.** Wt and IL-1R^−/−^ mice were inoculated intradermally with live or heat-killed *S. aureus* and real-time Q-PCR was performed on samples taken at 4 hrs after inoculation and from uninfected skin (n = 5 mice per group). (A) Real-time Q-PCR (mean fold change) after inoculation with either live or heat-killed *S. aureus* of the top 6 induced genes in wt mice (from Fig. 1A). Real-time Q-PCR (mean log_10_ fold change ± SEM) after inoculation with live *S. aureus* (B) or heat-killed *S. aureus* (C) of 2 representative genes that were similarly-induced and 8 representative genes that were differentially-induced in wt mice compared with IL-1R-deficient mice in the Cell Movement of Neutrophils sub-group from microarray analysis in Fig. 1D. The data in (B) is identical to data presented in Fig. 1E and is presented again in this figure so that the fold induction of these genes on the same scale can be directly compared between live (B) and heat-killed *S. aureus* (C).
(TIF)

Figure S3 **Other cell types that produce IL-1β at early time points after** ***S. aureus*** **skin infection.** pIL1-DsRed mice were infected intradermally with *S. aureus* and lesional skin specimens were collected at 4 hrs. Representative photomicrographs of sections labeled with anti-DsRed (IL-1β, red) and anti-CD45 (pan-leukocytes, green) (A) or anti-MHC II (antigen presenting cells, green) (B) and sections analyzed by confocal microscopy. Cells expressing both markers appear yellow (merge). High (left) and low (right) magnification images are shown (Scale bars = 50 mm). Dotted line = dermoepidermal junction. (C) Quantification of co-localization of IL-1β-DsRed fluorescence with CD45+ leukocytes or MHCII+ antigen presenting cells using the Manders' coefficient for a value range of 0 to 1 in which 0 = no pixels co-localize and 1 = all pixels co-localize. Data are representative from 4 mice per group.
(TIF)

Figure S4 **Additional representative confocal images of IL-1β expression in neutrophils and monocytes and isotype controls.** pIL1-DsRed mice were infected intradermally with *S. aureus* and lesional skin specimens were collected at 4 and 24 hrs. Representative photomicrographs of sections labeled with anti-DsRed (IL-1β, red) and anti-MOMA2 (monocytes/macrophages, green) (A) or anti-7/4 (neutrophils, green) (C) and sections analyzed by confocal microscopy. Cells expressing both markers appear yellow (merge). High (left) and low (right) magnification images are shown (Scale bars = 50 µm). Dotted line = dermoepidermal junction. (B, D) Representative photomicrographs of sections labeled with isotype control antibodies. Data are representative from 4 mice per group.
(TIF)

Figure S5 **7/4^+^ and MOMA2^+^ represent distinct cell types in** ***S. aureus*** **infected skin lesions.** pIL1-DsRed mice were infected intradermally with *S. aureus* and lesional skin specimens were collected at 4 and 24 hrs. Representative photomicrographs of sections labeled with anti-7/4 (neutrophils, green) and anti-MOMA2 (monocytes/macrophages, red) and sections analyzed by confocal microscopy. Cells expressing both markers appear yellow (merge). High (left) and low (right) magnification images are shown (Scale bars = 50 mm). Dotted line = dermoepidermal junction. Dotted line = dermoepidermal junction. Data are representative from 4 mice per group.
(TIF)

Figure S6 ***In vitro*** **functional assays for neutrophil function.** All assays were performed using anti-Ly6G bead-enriched neutrophils obtained from the bone marrow of wt or IL-1β-deficient mice. (A) Phagocytosis assay. Neutrophils were incubated with pHrodo-labeled *S. aureus* bioconjugates for 1 hr and internalization of bioconjugates was determined by flow cytometry. Data is represented as an average of pHrodo mean-fluorescence intensity (MFI) on gated neutrophils. (B) Degranulation assay. Neutrophils were stimulated for 30 minutes with 1 µM fMLF and lactoferrin release into the supernatant was measured by ELISA. (C) Oxidative burst assay. Neutrophils were stimulated for 30 minutes with 1 µM fMLF and the generation of reactive oxygen species was measured by flow cytometric analysis using a Phagoburst assay kit. Data is represented as a proportion of neutrophils that converted the substrate dihydrorhodamine-123 to fluorescent rhodamine-123. (D) Bacterial killing assay. Neutrophils were incubated with serum-opsonized *S. aureus* for 45 minutes. After incubation, neutrophils were diluted in H_2_O (pH 11) to lyse PMN, and serial dilutions were plated on TSB agar plates to enumerate viable bacterial CFU. Bacterial viability is expressed as percent viability relative to control wells without neutrophils. For all of these assays, data are from 3 wt or IL-1β-deficient mice per group. n.s. = not significant.
(TIF)

Figure S7 **Confirmation that neutrophils are the predominant source of IL-1β in the adoptive transfer experiment in** **Fig. 4** **.** (A) Neutrophils obtained after Percoll density gradient centrifugation of mouse bone marrow cells were labeled using mAbs specific for CD115 (clone AFS98) and Ly6G (clone 1A8) and analyzed by flow cytometry. The plot is representative of purity obtained from 3 different experiments. (B, C) Cells obtained after Percoll density gradient centrifugation of bone marrow cells from wt donor mice were first depleted with anti-Ly6G or anti-CD115 MACS bead separation (Miltenyi Biotec). Neutrophil or monocyte depleted cells were then adoptively transferred into IL-1β^−/−^ recipient mice. After 2 hrs, these mice and normal wt and IL-1β^−/−^ mice were infected intradermally with *S. aureus*. (B) Mean total lesion size (cm^2^) ± SEM. (C) *In vivo* bioluminescence quantified by mean total flux (photons/s) ± SEM (logarithmic scale). Data are from 6 mice per group. *p<0.05; ^†^p<0.01, ^‡^p<0.001, IL-1β^−/−^ mice or adoptively transferred mice versus wt mice (Student's *t*-test).
(TIF)

Figure S8 **IL-1β-expressing cells are found at the site of infection after adoptive-transfer of wt neutrophils into IL-1β-deficient mice.** Neutrophils from IL-1β^−/−^ or wt donor mice were adoptively transferred into IL-1β^−/−^ recipient mice. After 2 hrs, these mice and normal wt and IL-1β^−/−^ mice were infected intradermally with *S. aureus*. Representative photomicrographs of sections labeled with anti-IL-1β mAb (arrows) or isotype control mAb (immunoperoxidase method) of frozen sections of lesional skin at 1 day after skin inoculation with *S. aureus* (Scale bars = 50 µm). Data are representative of 3 mice per group. Scattered IL-1β-expressing cells are detected within the neutrophilic abscess of adoptively transferred of wt neutrophils but not IL-1β-deficient neutrophils into IL-1β^−/−^ mice after skin inoculation with *S. aureus*.
(TIF)

Figure S9 **Multiplicity of infection (MOI) and viability.** Neutrophils from mouse bone marrow were purified using anti-Ly6G MACS bead separation (Miltenyi Biotec) were infected with live *S. aureus* (SH1000) or MRSA (USA300 LAC strain) at a multiplicity of infection (MOI) of bacteria to neutrophils of 5∶1, 2∶1, 1∶1 or no bacteria for a total culture time of 6 hrs and gentamicin was added at 60 min from the start of the infection to prevent bacterial overgrowth. Data are from neutrophils obtained from 5 mice per group. (A, B) IL-1β protein levels (mean ± SEM) were measured in culture supernatants by ELISA. (C, D) Cell viability of the neutrophils in infected and uninfected cultures was measured using a viability assay kit (Promega, Madison, WI). Data presented as the percent viability (mean ± SEM) of *S. aureus*-infected (C) or MRSA-infected (D) neutrophils at the different MOI compared with the viability of uninfected neutrophils.
(TIF)

Figure S10 **Neutrophils were the primary cells that had IL-1β-promoter activity after** ***in vitro*** **infection with** ***S. aureus*** **.** Mouse neutrophils were obtained from bone marrow cells of pIL1-DsRed mice using anti-Ly6G MACS beads [Miltenyi Biotec]). Under the same culture conditions as in Fig. 6, these neutrophils were infected with live *S. aureus* (SH1000) at an MOI of bacteria to neutrophils of 5∶1 or no bacteria (uninfected) for a total culture time of 6 hrs and gentamicin was added at 60 min from the start of the infection to prevent bacterial overgrowth. After 6 hrs of culture the neutrophils were harvested and labeled using mAbs specific for CD115 (clone AFS98) and Ly6G (clone 1A8) and analyzed by flow cytometry. Plots are representative of 3 different experiments. After *S. aureus in vitro* infection, 43% of the Ly6G^+^ CD115^−^ neutrophils had IL-1β-DsRed fluorescence whereas only 0.2% that DsRed^+^ Ly6G^−^ cells had IL-1β-DsRed fluorescence, indicating that neutrophils represented almost all of the cells that had IL-1β-promoter activity during *S. aureus in vitro* infection.
(TIF)

Figure S11 **Viability of mouse neutrophils for the** ***in vitro*** **infection experiments in** **Fig. 6C and D** **.** Neutrophils from mouse bone marrow were infected with live *S. aureus* (SH1000) or MRSA (USA300 LAC strain) (MOI bacteria∶neutrophils of 5∶1) for a total culture time of 6 hrs and gentamicin was added at 60 min from the start of the infection to prevent bacterial overgrowth. (A, B) Cell viability of the neutrophils infected with (A) *S. aureus* or (B) MRSA in the presence or absence of an NLRP3-inhibitor (glibenclamide), a caspase-1 inhibitor (Z-YVAD-FMK) or anti-staphylococcal α-toxin antibodies was measured using a viability assay kit (Promega, Madison, WI). Data presented as the percent viability (mean ± SEM) compared with the viability of uninfected neutrophils. Data are from 3 mice per group.
(TIF)

Figure S12 **Immunoblotting for detection of pro-IL-1β and cleaved IL-1β.** Neutrophils from mouse bone marrow were purified using anti-Ly6G MACS bead separation (Miltenyi Biotec) were infected with live *S. aureus* (SH1000) or MRSA (USA300 LAC strain) (MOI bacteria∶neutrophils of 5∶1) for a total culture time of 6 hrs and gentamicin was added at 60 min from the start of the infection to prevent bacterial overgrowth. Pro-IL-1β protein (35 kDa) and cleaved IL-1β protein (17 kDa) was detected by immunoblot of cell lysates using a polyclonal antibody against IL-1β. Cleaved IL-1β protein was only detected in *S. aureus*- or MRSA-infected neutrophils but not in uninfected neutrophils.
(TIF)

Figure S13 **Purity of mouse neutrophils for the** ***in vitro*** **infection experiments in** **Fig. 6** **.** (A) The purity of the of mouse neutrophils obtained from bone marrow cells using anti-Ly6G MACS bead separation [Miltenyi Biotec]) for the *in vitro* infection experiments in Fig. 6 was determined by labeling the cells with mAbs specific for Ly6G (clone 1A8) and CD11b (clone M1/70) and by flow cytometry analysis. Plots are representative of mouse neutrophil purity from 3 different experiments. There were 99.1% Ly6G^+^ CD11b^high^ neutrophils. There were very few Ly6G^−^ CD11b^low^ monocytes (0.1%) and the remaining cells (0.6%) were Ly6G^−^ CD11b^−^ cells.
(TIF)

Figure S14 **Adoptive transfer of wt neutrophils is not sufficient to rescue TLR2-deficient mice.** Neutrophils from wt donor mice were adoptively transferred into TLR2^−/−^ recipient mice. After 2 hrs, these mice and normal wt and TLR2^−/−^ mice were infected intradermally with *S. aureus*. (A) Mean total lesion size (cm^2^) ± SEM. (B) *In vivo* bioluminescence quantified by mean total flux (photons/s) ± SEM (logarithmic scale). Data is representative of at least 4 mice per group. *p<0.05; TLR2^−/−^ mice or TLR2^−/−^ mice with adoptively transferred wt neutrophils versus wt mice (Student's t-test).
(TIF)

Figure S15 **IL-1β protein expression in keratinocytes overlying the** ***S. aureus*** **infection in the dermis.** Wt mice were infected intradermally with *S. aureus* and lesional skin specimens were collected at 24 hrs. Representative photomicrographs of sections labeled with anti-IL-1β mAb or isotype control mAb (immunoperoxidase method) of frozen sections of lesional skin at 1 day after skin inoculation with *S. aureus* (Scale bars = 50 µm). Data are representative of 3 different wt mice. IL-1β was found to be expressed within some of the epidermal keratinocytes overlying the abscess after skin inoculation with *S. aureus*.
(TIF)

Figure S16 **Cellular composition in the** ***S. aureus*** **-infected skin at day 10 after infection.** TLR2-, NOD2-, FPR1-, and IL-1β-deficient mice as well as wt mice were inoculated intradermally with *S. aureus* and lesional skin specimens were collected at 10 days after infection. Representative photomicrographs of sections labeled with anti-7/4 (neutrophils) or anti-MOMA2 (monocytes/macrophages) or isotype control mAb (immunoperoxidase method) of frozen sections lesional skin at 10 days after skin inoculation with *S. aureus* (Scale bars = 100 µm). Data is representative of 3 mice per group. The cellular composition of 7/4^+^ neutrophils and MOMA2^+^ monocytes/macrophages on day 10 in TLR2-, NOD2-, FPR1-deficient mice after infection was similar to the cellular composition in wt mice whereas IL-1β-deficient mice had a paucity of 7/4^+^ neutrophils at this time point.
(TIF)

Figure S17 **Localization of EGFP in neutrophils from LysEGFP mice.** (A) Neutrophils from LysEGFP mice labeled with Hoescht 33342 counterstain, mounted on microscope slides and imaged with an Olympus BX61 fluorescence microscope (100× objective). Green = EGFP and Blue = nucleus. Scale bar = 10 µm. (B) Mouse neutrophils enriched from bone marrow of LysEGFP mice were left unstimulated or stimulated with fMLF or PMA and EGFP fluorescent signals and CD11b expression (as an positive marker for neutrophil activation) were evaluated using flow cytometry. Neutrophils were first gated on forward and side scatter and mature neutrophils were then identified by high Ly6G^+^ expression. Mean fluorescence intensity (MFI) of EGFP and CD11b on neutrophils is indicated for unstimulated and stimulated neutrophils. Data are representative from 3 different LysEGFP mice.
(TIF)
