# Supplementary figures and images for: Neutrophil-derived IL-1β Is Sufficient for Abscess Formation in Immunity against Staphylococcus aureus in Mice
Source: PLoS Pathog. 2012 Nov 29;8(11):e1003047. doi: 10.1371/journal.ppat.1003047 (PMC3510260; doi:10.1371/journal.ppat.1003047)

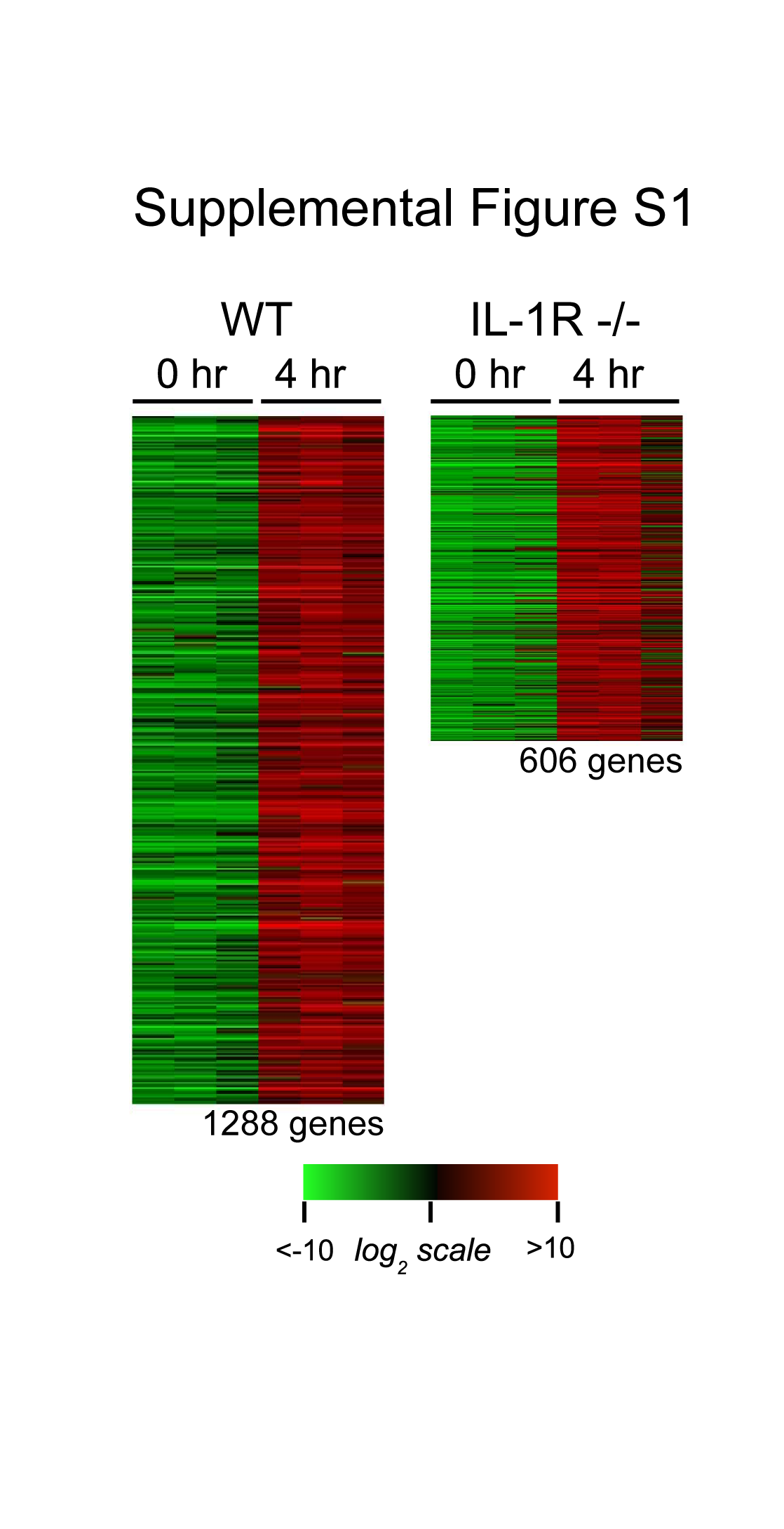

Supplement: Figure S1 — Differentially expressed genes. Genes differentially expressed between 0 and 4 hrs post-infection were identified according to the following criteria: fold change >1.5 and p-value<0.05. The heatmap was generated using the R statistical package (www.r-project.org). (TIF) [file ppat.1003047.s001.tif]

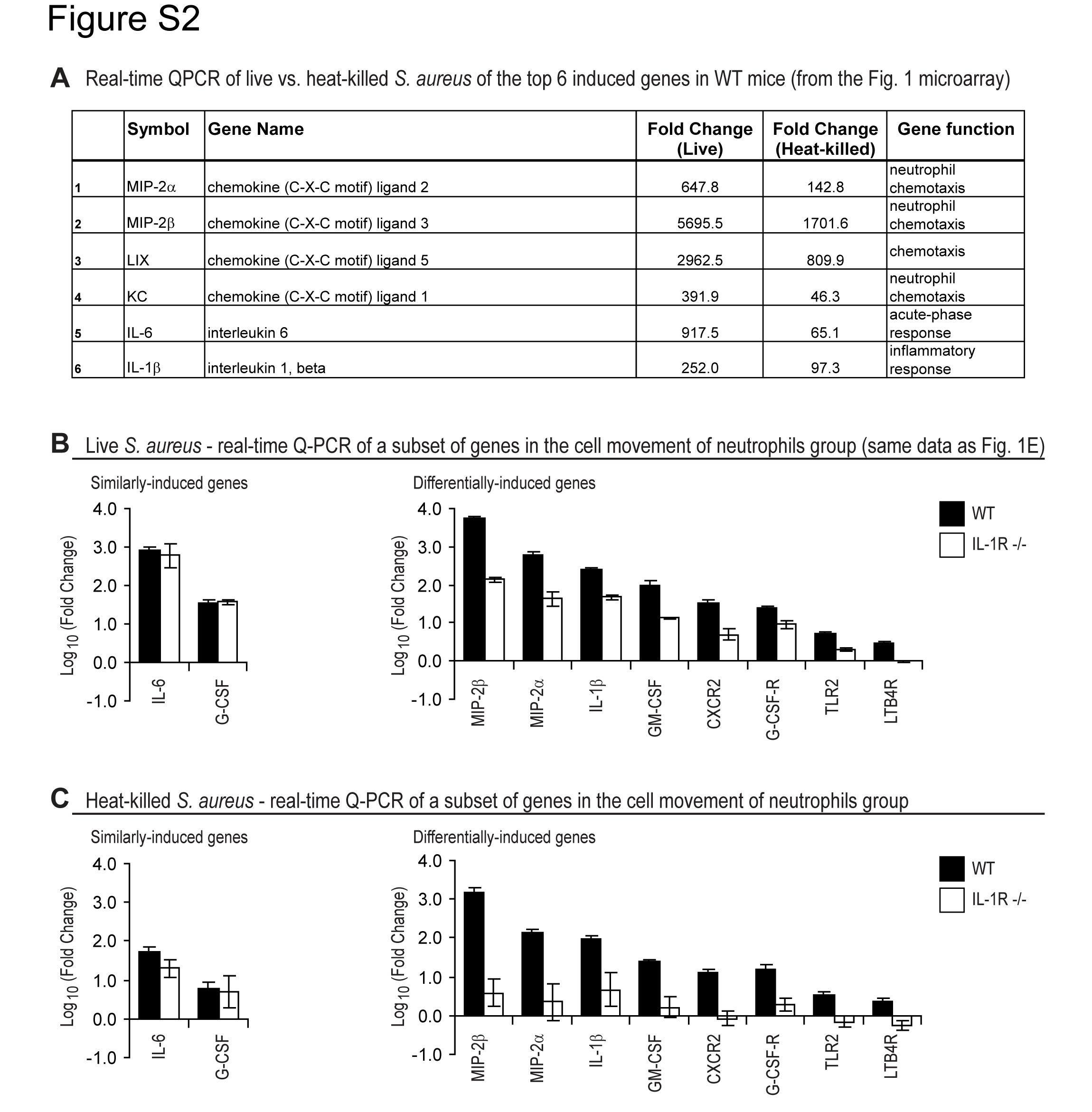

Supplement: Figure S2 — Comparison of gene expression between inoculation of live and heat-killed S. aureus in the skin of wt versus IL-1R-deficient mice. Wt and IL-1R−/− mice were inoculated intradermally with live or heat-killed S. aureus and real-time Q-PCR was performed on samples taken at 4 hrs after inoculation and from uninfected skin (n = 5 mice per group). (A) Real-time Q-PCR (mean fold change) after inoculation with either live or heat-killed S. aureus of the top 6 induced genes in wt mice (from Fig. 1A). Real-time Q-PCR (mean log10 fold change ± SEM) after inoculation with live S. aureus (B) or heat-killed S. aureus (C) of 2 representative genes that were similarly-induced and 8 representative genes that were differentially-induced in wt mice compared with IL-1R-deficient mice in the Cell Movement of Neutrophils sub-group from microarray analysis in Fig. 1D. The data in (B) is identical to data presented in Fig. 1E and is presented again in this figure so that the fold induction of these genes on the same scale can be directly compared between live (B) and heat-killed S. aureus (C). (TIF) [file ppat.1003047.s002.tif]

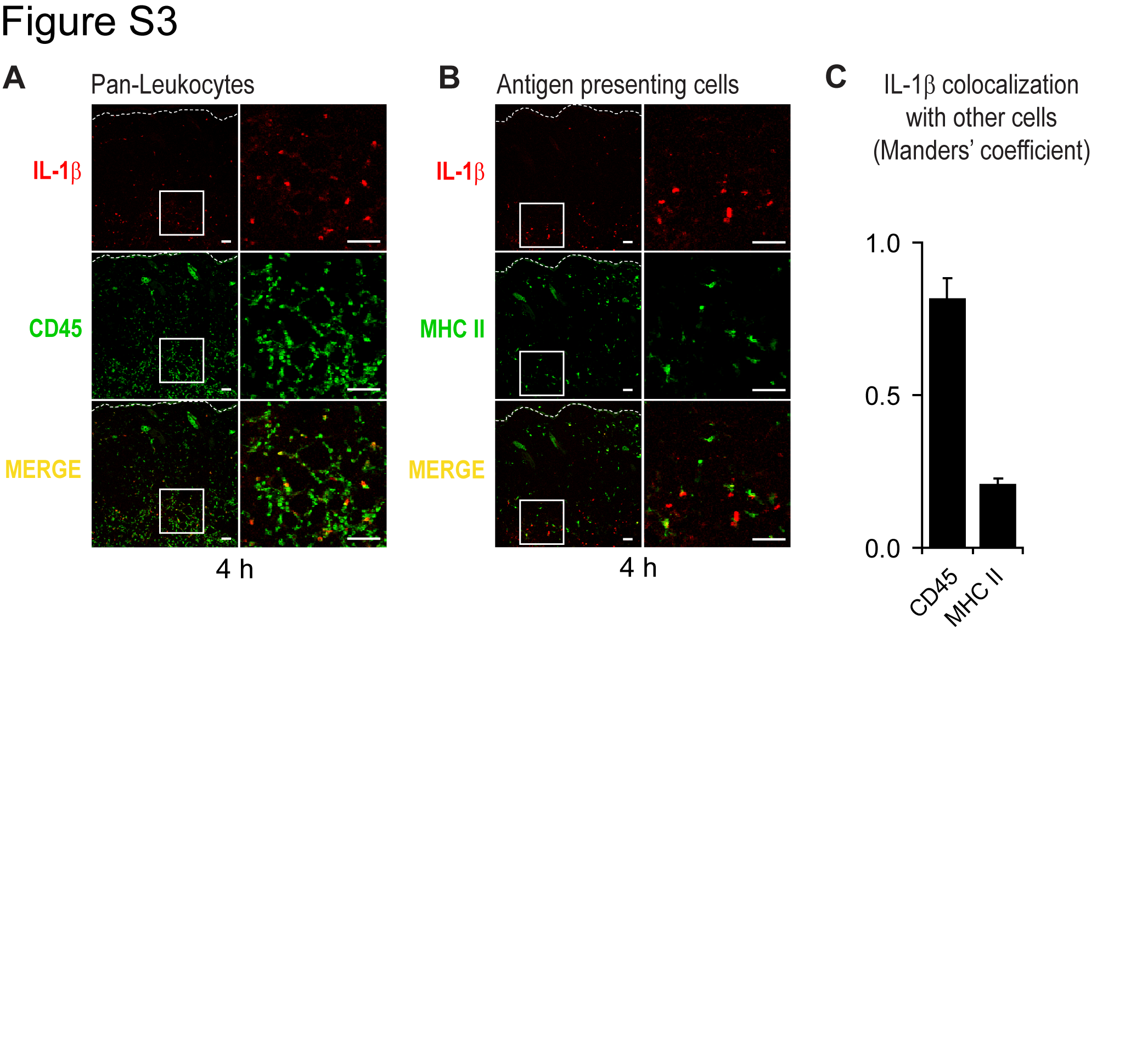

Supplement: Figure S3 — Other cell types that produce IL-1β at early time points after S. aureus skin infection. pIL1-DsRed mice were infected intradermally with S. aureus and lesional skin specimens were collected at 4 hrs. Representative photomicrographs of sections labeled with anti-DsRed (IL-1β, red) and anti-CD45 (pan-leukocytes, green) (A) or anti-MHC II (antigen presenting cells, green) (B) and sections analyzed by confocal microscopy. Cells expressing both markers appear yellow (merge). High (left) and low (right) magnification images are shown (Scale bars = 50 mm). Dotted line = dermoepidermal junction. (C) Quantification of co-localization of IL-1β-DsRed fluorescence with CD45+ leukocytes or MHCII+ antigen presenting cells using the Manders' coefficient for a value range of 0 to 1 in which 0 = no pixels co-localize and 1 = all pixels co-localize. Data are representative from 4 mice per group. (TIF) [file ppat.1003047.s003.tif]

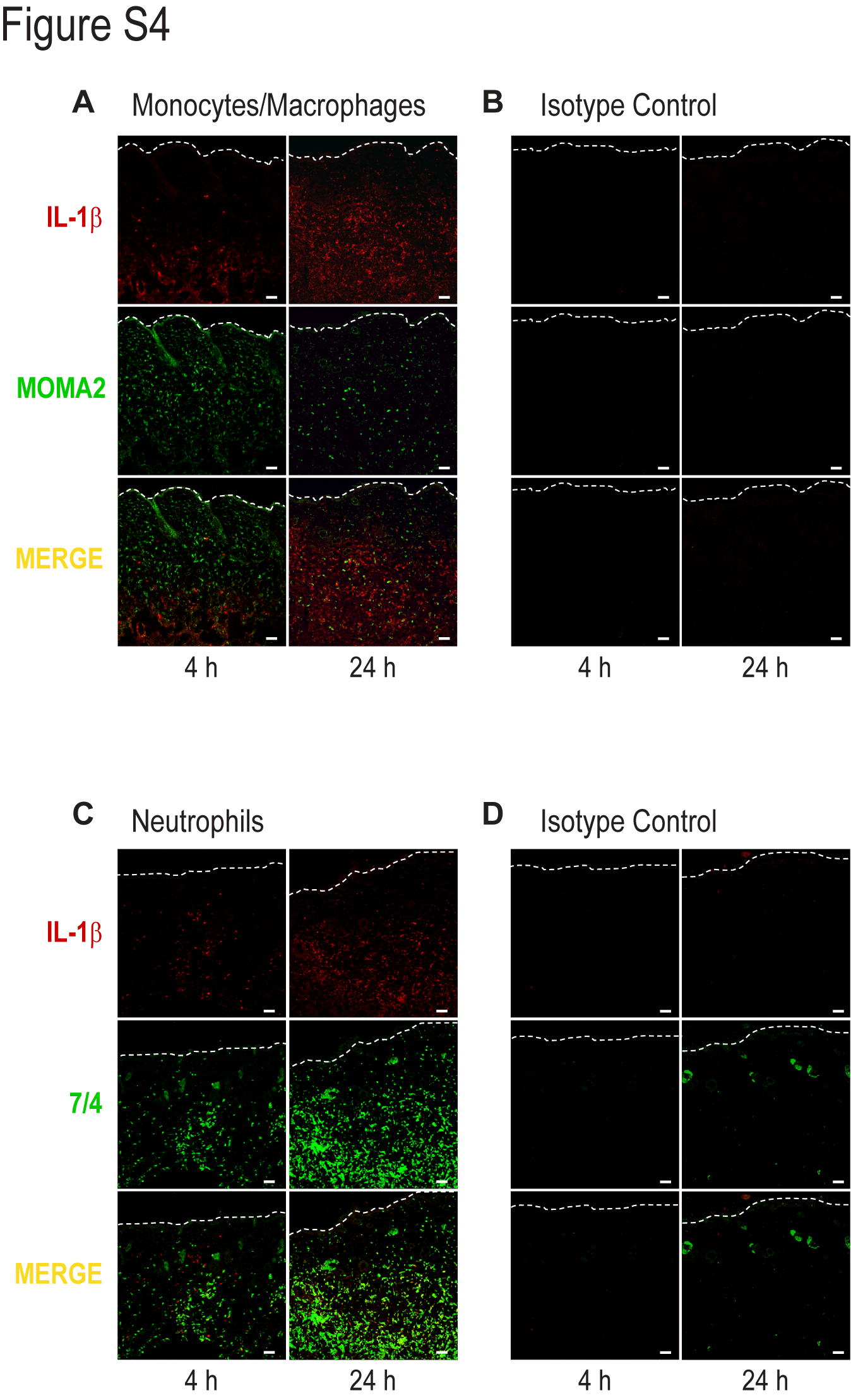

Supplement: Figure S4 — Additional representative confocal images of IL-1β expression in neutrophils and monocytes and isotype controls. pIL1-DsRed mice were infected intradermally with S. aureus and lesional skin specimens were collected at 4 and 24 hrs. Representative photomicrographs of sections labeled with anti-DsRed (IL-1β, red) and anti-MOMA2 (monocytes/macrophages, green) (A) or anti-7/4 (neutrophils, green) (C) and sections analyzed by confocal microscopy. Cells expressing both markers appear yellow (merge). High (left) and low (right) magnification images are shown (Scale bars = 50 µm). Dotted line = dermoepidermal junction. (B, D) Representative photomicrographs of sections labeled with isotype control antibodies. Data are representative from 4 mice per group. (TIF) [file ppat.1003047.s004.tif]

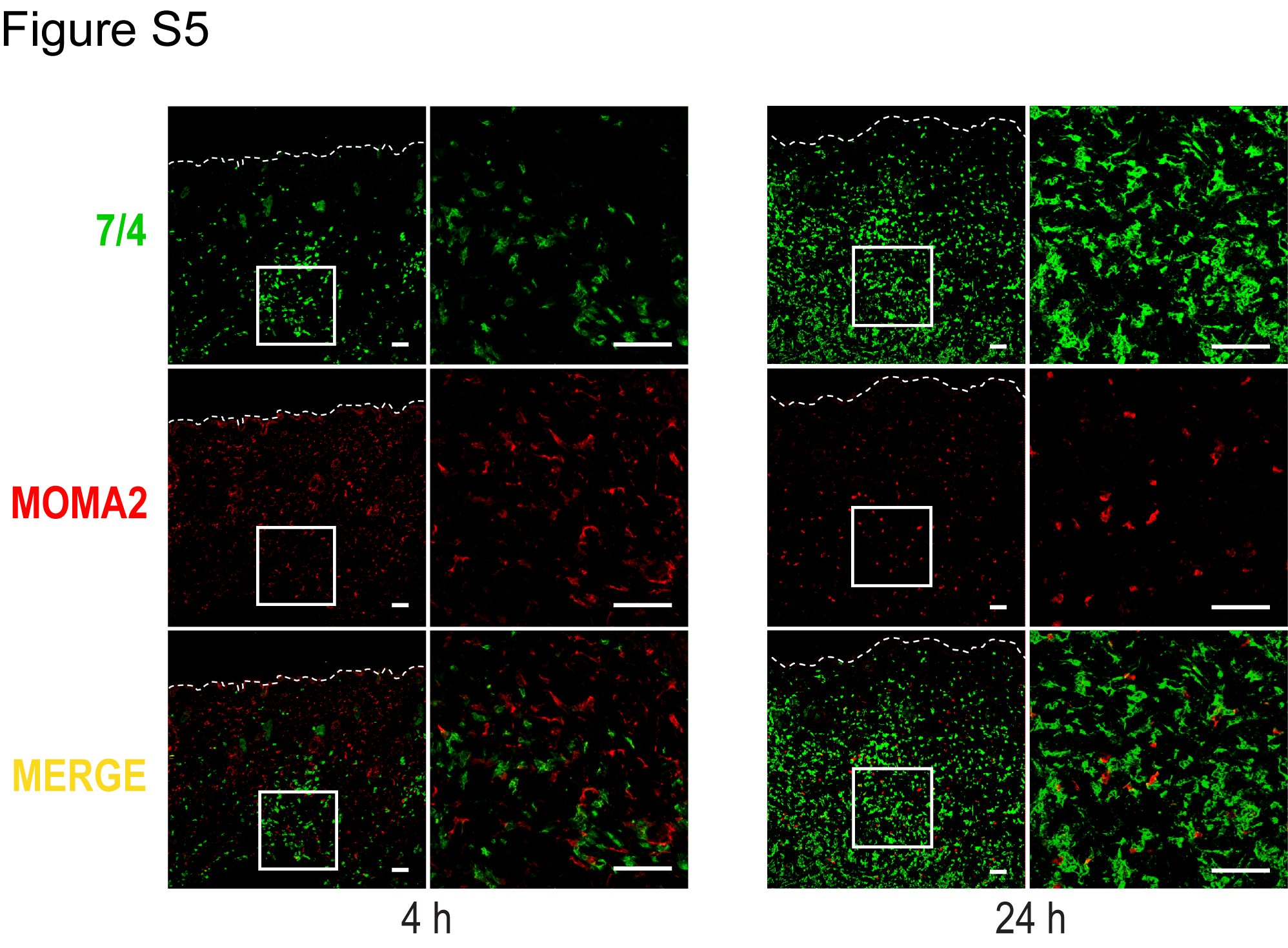

Supplement: Figure S5 — 7/4+ and MOMA2+ represent distinct cell types in S. aureus infected skin lesions. pIL1-DsRed mice were infected intradermally with S. aureus and lesional skin specimens were collected at 4 and 24 hrs. Representative photomicrographs of sections labeled with anti-7/4 (neutrophils, green) and anti-MOMA2 (monocytes/macrophages, red) and sections analyzed by confocal microscopy. Cells expressing both markers appear yellow (merge). High (left) and low (right) magnification images are shown (Scale bars = 50 mm). Dotted line = dermoepidermal junction. Dotted line = dermoepidermal junction. Data are representative from 4 mice per group. (TIF) [file ppat.1003047.s005.tif]

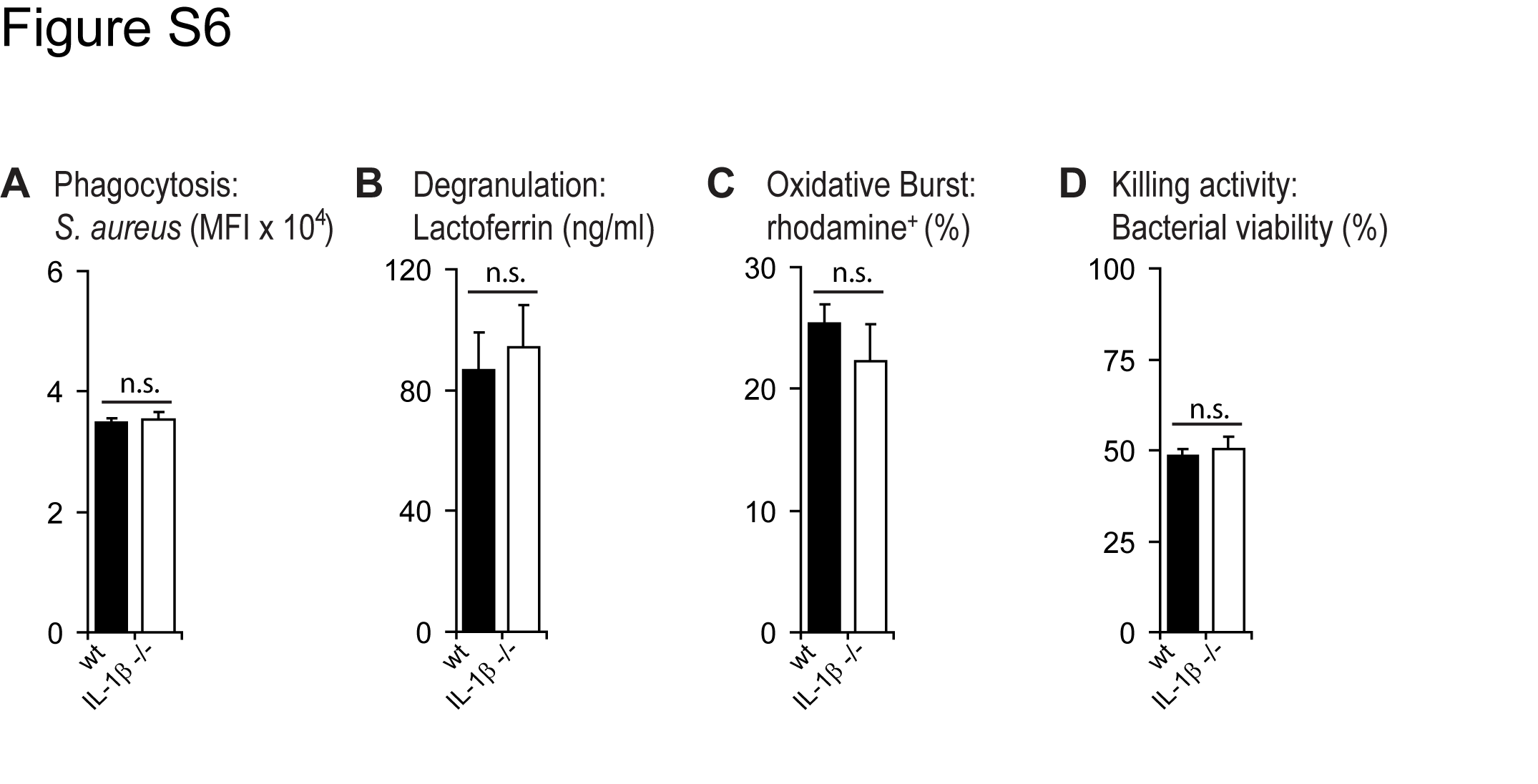

Supplement: Figure S6 — In vitro functional assays for neutrophil function. All assays were performed using anti-Ly6G bead-enriched neutrophils obtained from the bone marrow of wt or IL-1β-deficient mice. (A) Phagocytosis assay. Neutrophils were incubated with pHrodo-labeled S. aureus bioconjugates for 1 hr and internalization of bioconjugates was determined by flow cytometry. Data is represented as an average of pHrodo mean-fluorescence intensity (MFI) on gated neutrophils. (B) Degranulation assay. Neutrophils were stimulated for 30 minutes with 1 µM fMLF and lactoferrin release into the supernatant was measured by ELISA. (C) Oxidative burst assay. Neutrophils were stimulated for 30 minutes with 1 µM fMLF and the generation of reactive oxygen species was measured by flow cytometric analysis using a Phagoburst assay kit. Data is represented as a proportion of neutrophils that converted the substrate dihydrorhodamine-123 to fluorescent rhodamine-123. (D) Bacterial killing assay. Neutrophils were incubated with serum-opsonized S. aureus for 45 minutes. After incubation, neutrophils were diluted in H2O (pH 11) to lyse PMN, and serial dilutions were plated on TSB agar plates to enumerate viable bacterial CFU. Bacterial viability is expressed as percent viability relative to control wells without neutrophils. For all of these assays, data are from 3 wt or IL-1β-deficient mice per group. n.s. = not significant. (TIF) [file ppat.1003047.s006.tif]

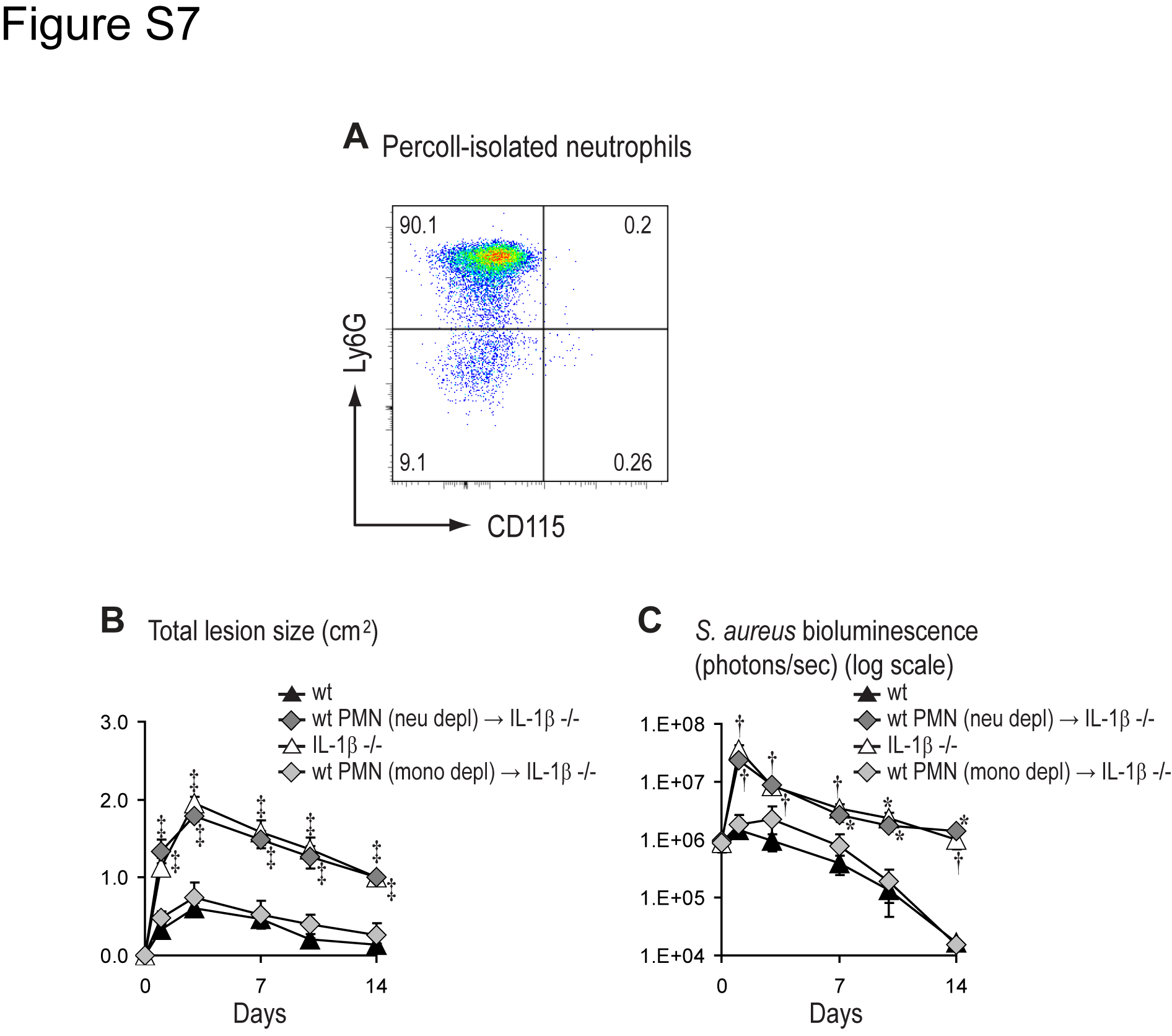

Supplement: Figure S7 — Confirmation that neutrophils are the predominant source of IL-1β in the adoptive transfer experiment in Fig. 4 . (A) Neutrophils obtained after Percoll density gradient centrifugation of mouse bone marrow cells were labeled using mAbs specific for CD115 (clone AFS98) and Ly6G (clone 1A8) and analyzed by flow cytometry. The plot is representative of purity obtained from 3 different experiments. (B, C) Cells obtained after Percoll density gradient centrifugation of bone marrow cells from wt donor mice were first depleted with anti-Ly6G or anti-CD115 MACS bead separation (Miltenyi Biotec). Neutrophil or monocyte depleted cells were then adoptively transferred into IL-1β−/− recipient mice. After 2 hrs, these mice and normal wt and IL-1β−/− mice were infected intradermally with S. aureus. (B) Mean total lesion size (cm2) ± SEM. (C) In vivo bioluminescence quantified by mean total flux (photons/s) ± SEM (logarithmic scale). Data are from 6 mice per group. *p<0.05; †p<0.01, ‡p<0.001, IL-1β−/− mice or adoptively transferred mice versus wt mice (Student's t-test). (TIF) [file ppat.1003047.s007.tif]

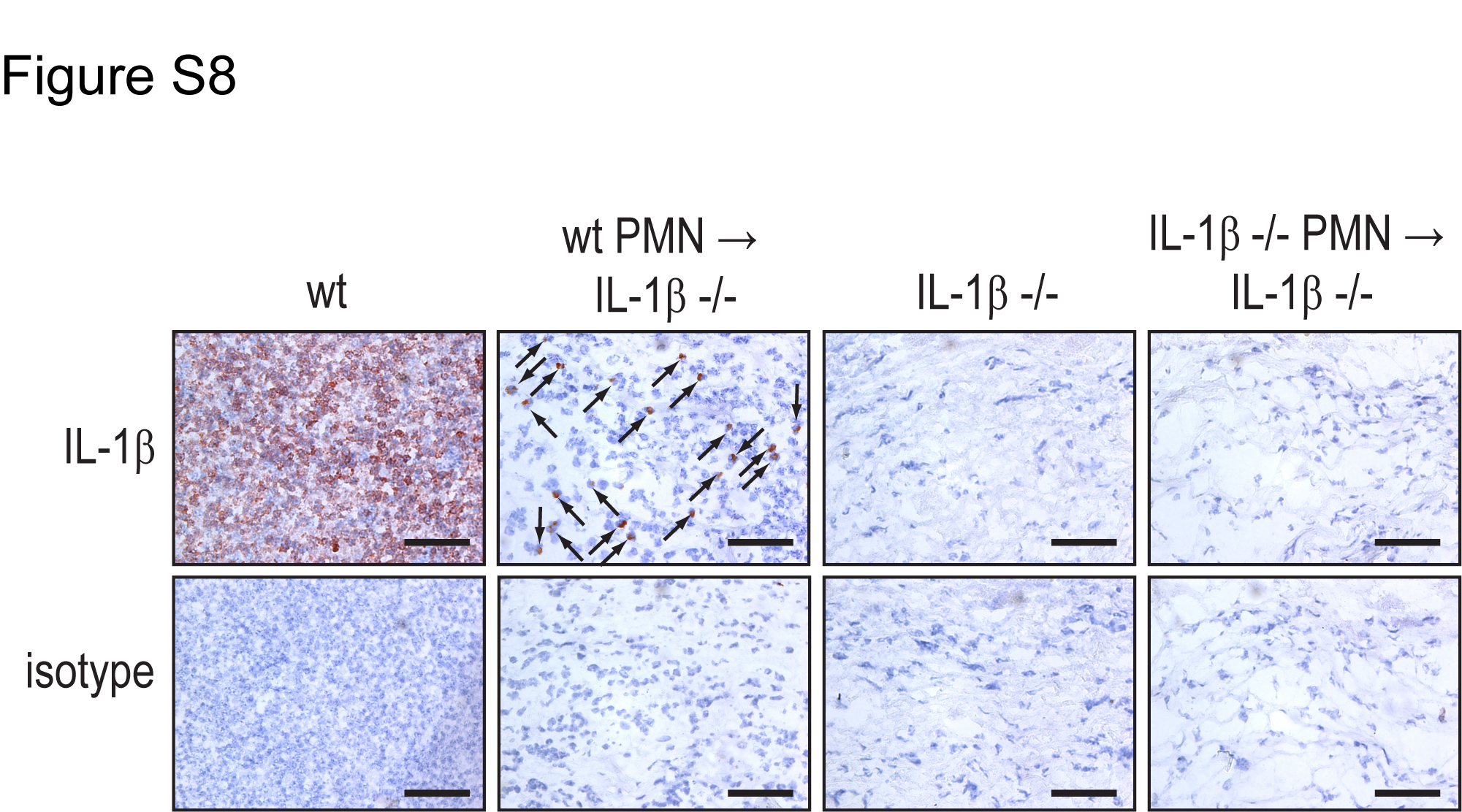

Supplement: Figure S8 — IL-1β-expressing cells are found at the site of infection after adoptive-transfer of wt neutrophils into IL-1β-deficient mice. Neutrophils from IL-1β−/− or wt donor mice were adoptively transferred into IL-1β−/− recipient mice. After 2 hrs, these mice and normal wt and IL-1β−/− mice were infected intradermally with S. aureus. Representative photomicrographs of sections labeled with anti-IL-1β mAb (arrows) or isotype control mAb (immunoperoxidase method) of frozen sections of lesional skin at 1 day after skin inoculation with S. aureus (Scale bars = 50 µm). Data are representative of 3 mice per group. Scattered IL-1β-expressing cells are detected within the neutrophilic abscess of adoptively transferred of wt neutrophils but not IL-1β-deficient neutrophils into IL-1β−/− mice after skin inoculation with S. aureus. (TIF) [file ppat.1003047.s008.tif]

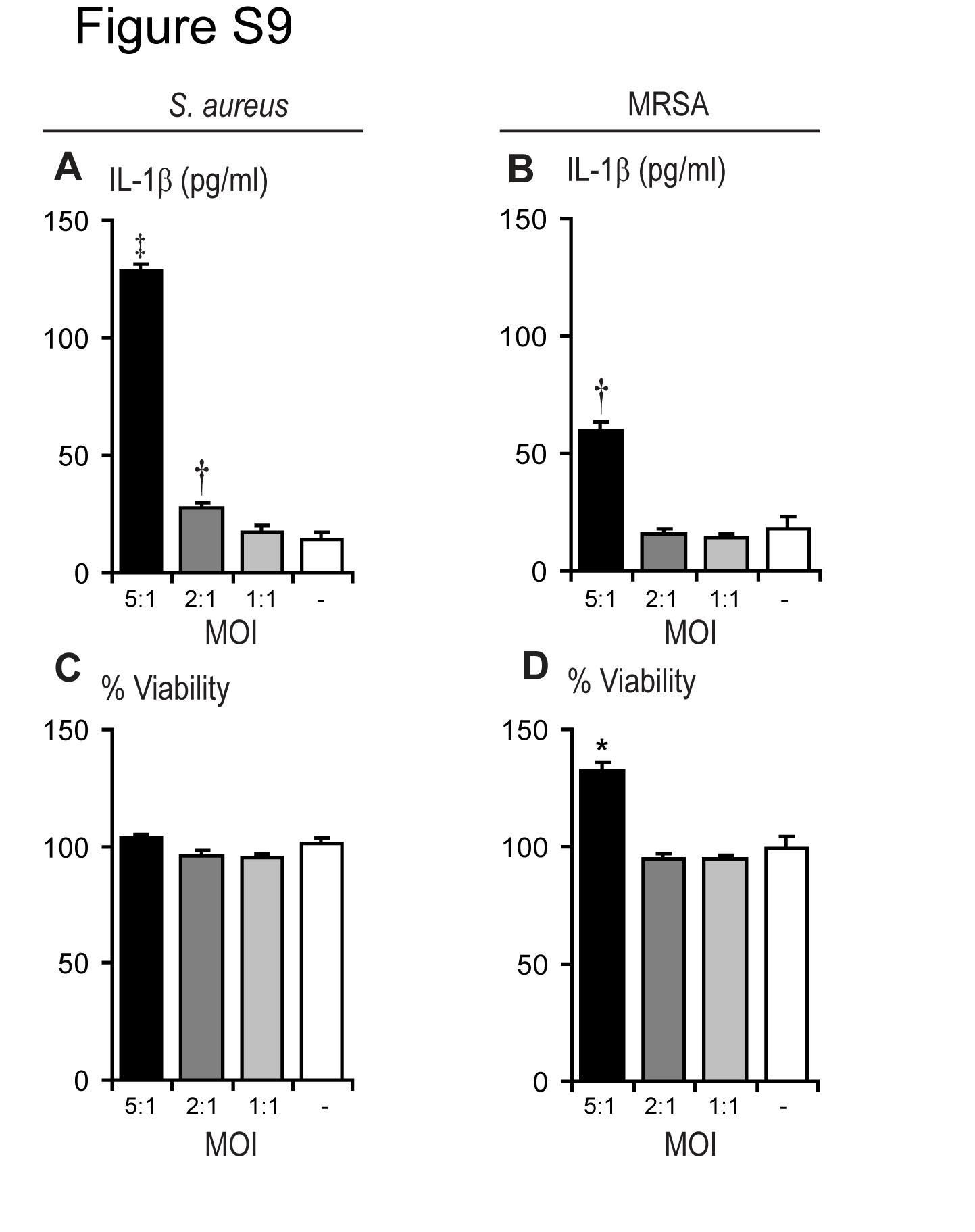

Supplement: Figure S9 — Multiplicity of infection (MOI) and viability. Neutrophils from mouse bone marrow were purified using anti-Ly6G MACS bead separation (Miltenyi Biotec) were infected with live S. aureus (SH1000) or MRSA (USA300 LAC strain) at a multiplicity of infection (MOI) of bacteria to neutrophils of 5∶1, 2∶1, 1∶1 or no bacteria for a total culture time of 6 hrs and gentamicin was added at 60 min from the start of the infection to prevent bacterial overgrowth. Data are from neutrophils obtained from 5 mice per group. (A, B) IL-1β protein levels (mean ± SEM) were measured in culture supernatants by ELISA. (C, D) Cell viability of the neutrophils in infected and uninfected cultures was measured using a viability assay kit (Promega, Madison, WI). Data presented as the percent viability (mean ± SEM) of S. aureus-infected (C) or MRSA-infected (D) neutrophils at the different MOI compared with the viability of uninfected neutrophils. (TIF) [file ppat.1003047.s009.tif]

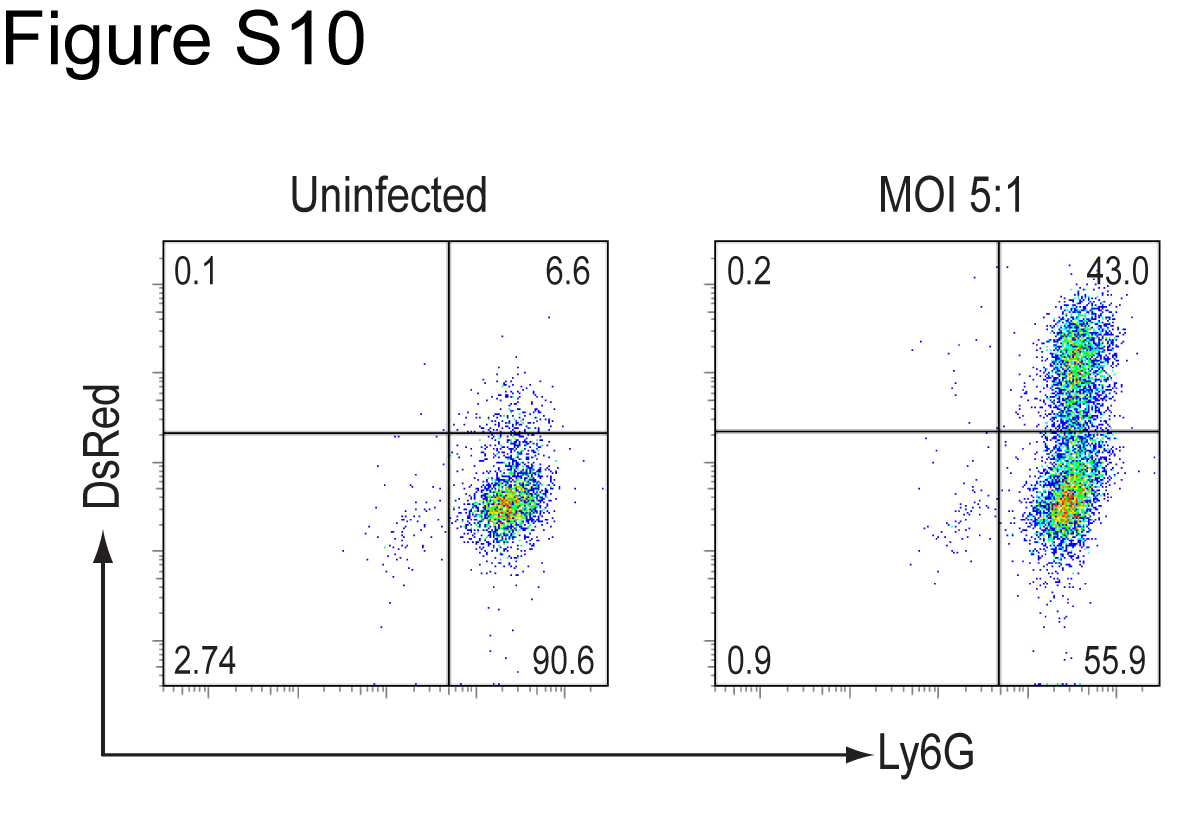

Supplement: Figure S10 — Neutrophils were the primary cells that had IL-1β-promoter activity after in vitro infection with S. aureus . Mouse neutrophils were obtained from bone marrow cells of pIL1-DsRed mice using anti-Ly6G MACS beads [Miltenyi Biotec]). Under the same culture conditions as in Fig. 6, these neutrophils were infected with live S. aureus (SH1000) at an MOI of bacteria to neutrophils of 5∶1 or no bacteria (uninfected) for a total culture time of 6 hrs and gentamicin was added at 60 min from the start of the infection to prevent bacterial overgrowth. After 6 hrs of culture the neutrophils were harvested and labeled using mAbs specific for CD115 (clone AFS98) and Ly6G (clone 1A8) and analyzed by flow cytometry. Plots are representative of 3 different experiments. After S. aureus in vitro infection, 43% of the Ly6G+ CD115− neutrophils had IL-1β-DsRed fluorescence whereas only 0.2% that DsRed+ Ly6G− cells had IL-1β-DsRed fluorescence, indicating that neutrophils represented almost all of the cells that had IL-1β-promoter activity during S. aureus in vitro infection. (TIF) [file ppat.1003047.s010.tif]

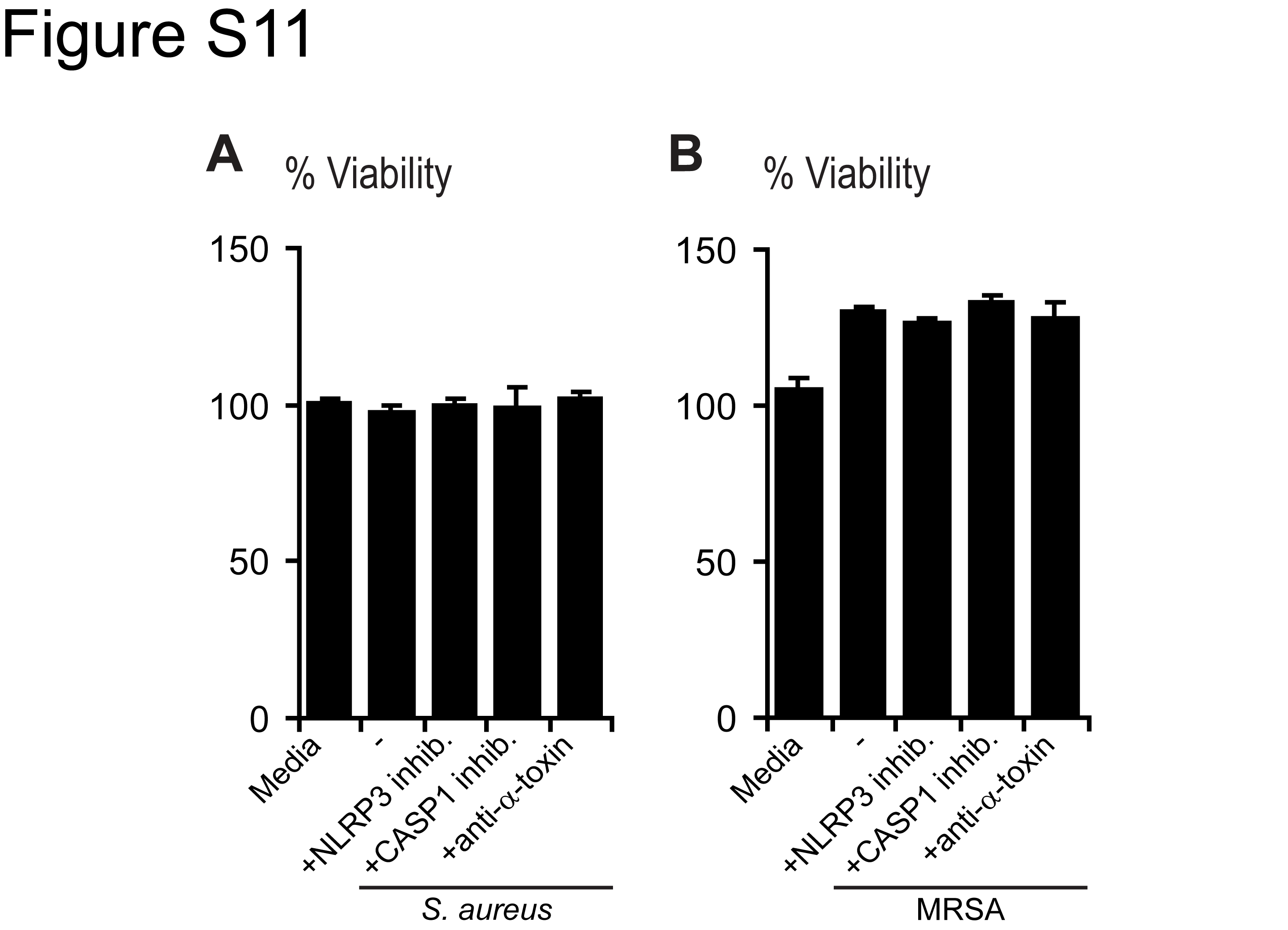

Supplement: Figure S11 — Viability of mouse neutrophils for the in vitro infection experiments in Fig. 6C and D . Neutrophils from mouse bone marrow were infected with live S. aureus (SH1000) or MRSA (USA300 LAC strain) (MOI bacteria∶neutrophils of 5∶1) for a total culture time of 6 hrs and gentamicin was added at 60 min from the start of the infection to prevent bacterial overgrowth. (A, B) Cell viability of the neutrophils infected with (A) S. aureus or (B) MRSA in the presence or absence of an NLRP3-inhibitor (glibenclamide), a caspase-1 inhibitor (Z-YVAD-FMK) or anti-staphylococcal α-toxin antibodies was measured using a viability assay kit (Promega, Madison, WI). Data presented as the percent viability (mean ± SEM) compared with the viability of uninfected neutrophils. Data are from 3 mice per group. (TIF) [file ppat.1003047.s011.tif]

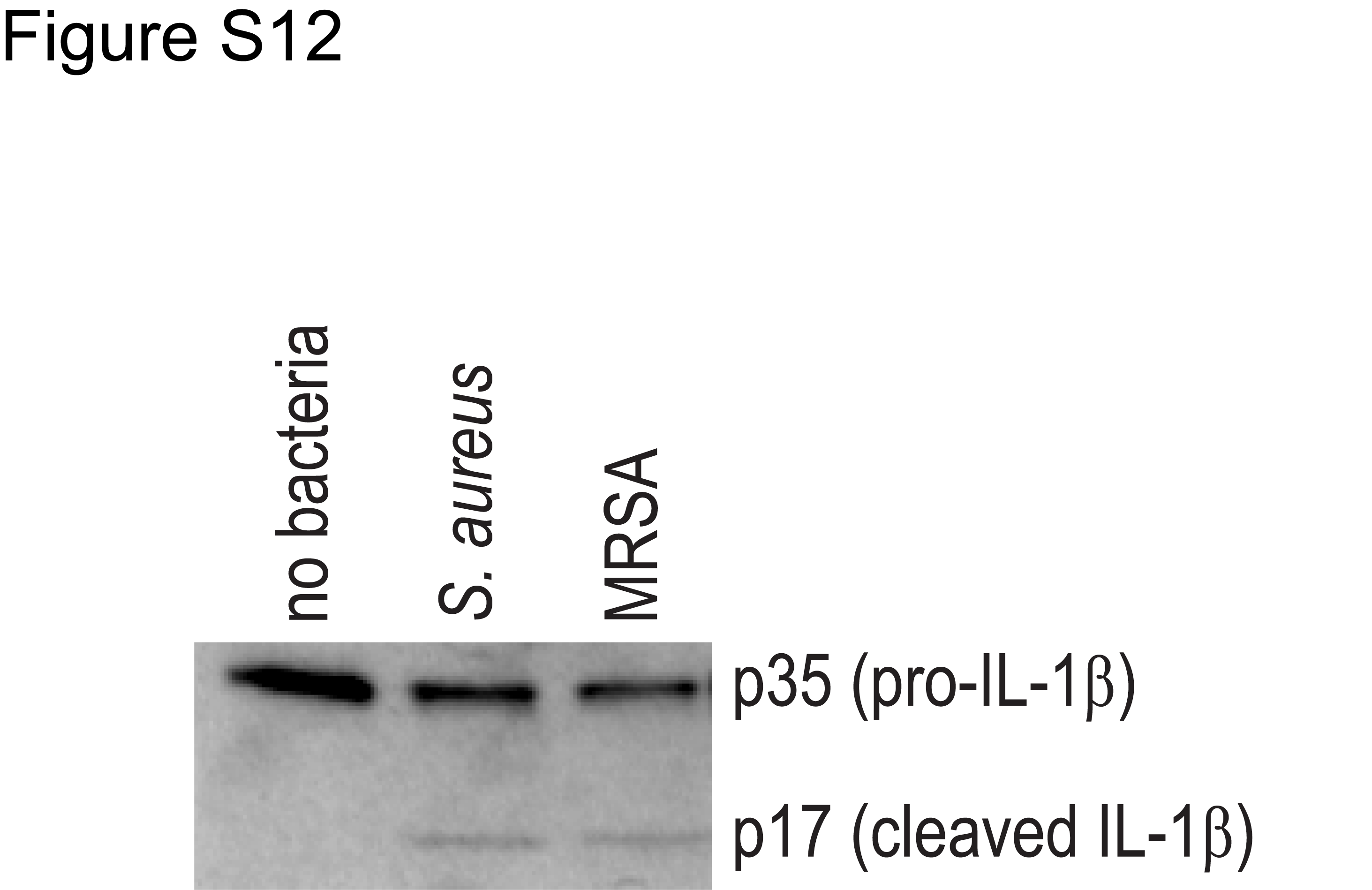

Supplement: Figure S12 — Immunoblotting for detection of pro-IL-1β and cleaved IL-1β. Neutrophils from mouse bone marrow were purified using anti-Ly6G MACS bead separation (Miltenyi Biotec) were infected with live S. aureus (SH1000) or MRSA (USA300 LAC strain) (MOI bacteria∶neutrophils of 5∶1) for a total culture time of 6 hrs and gentamicin was added at 60 min from the start of the infection to prevent bacterial overgrowth. Pro-IL-1β protein (35 kDa) and cleaved IL-1β protein (17 kDa) was detected by immunoblot of cell lysates using a polyclonal antibody against IL-1β. Cleaved IL-1β protein was only detected in S. aureus- or MRSA-infected neutrophils but not in uninfected neutrophils. (TIF) [file ppat.1003047.s012.tif]

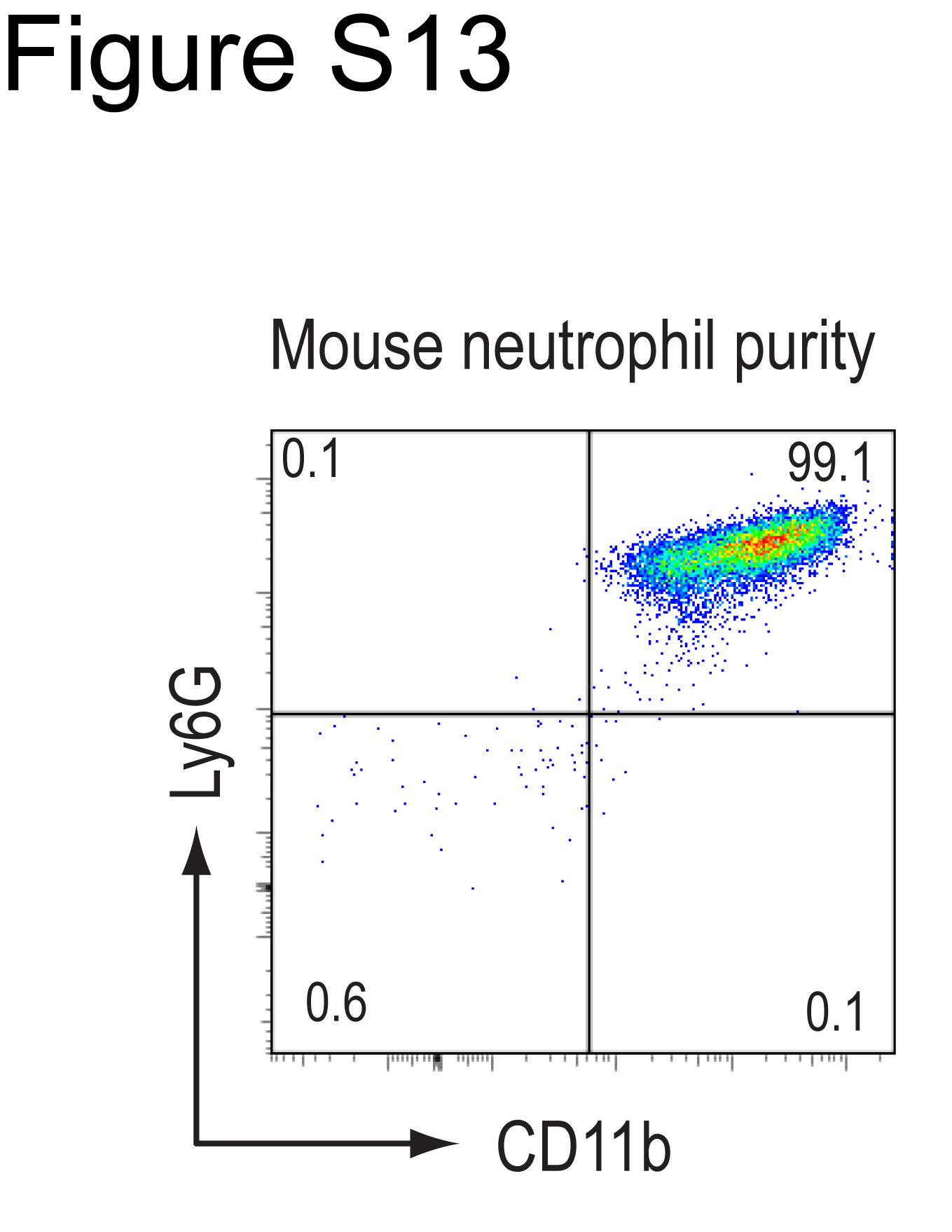

Supplement: Figure S13 — Purity of mouse neutrophils for the in vitro infection experiments in Fig. 6 . (A) The purity of the of mouse neutrophils obtained from bone marrow cells using anti-Ly6G MACS bead separation [Miltenyi Biotec]) for the in vitro infection experiments in Fig. 6 was determined by labeling the cells with mAbs specific for Ly6G (clone 1A8) and CD11b (clone M1/70) and by flow cytometry analysis. Plots are representative of mouse neutrophil purity from 3 different experiments. There were 99.1% Ly6G+ CD11bhigh neutrophils. There were very few Ly6G− CD11blow monocytes (0.1%) and the remaining cells (0.6%) were Ly6G− CD11b− cells. (TIF) [file ppat.1003047.s013.tif]

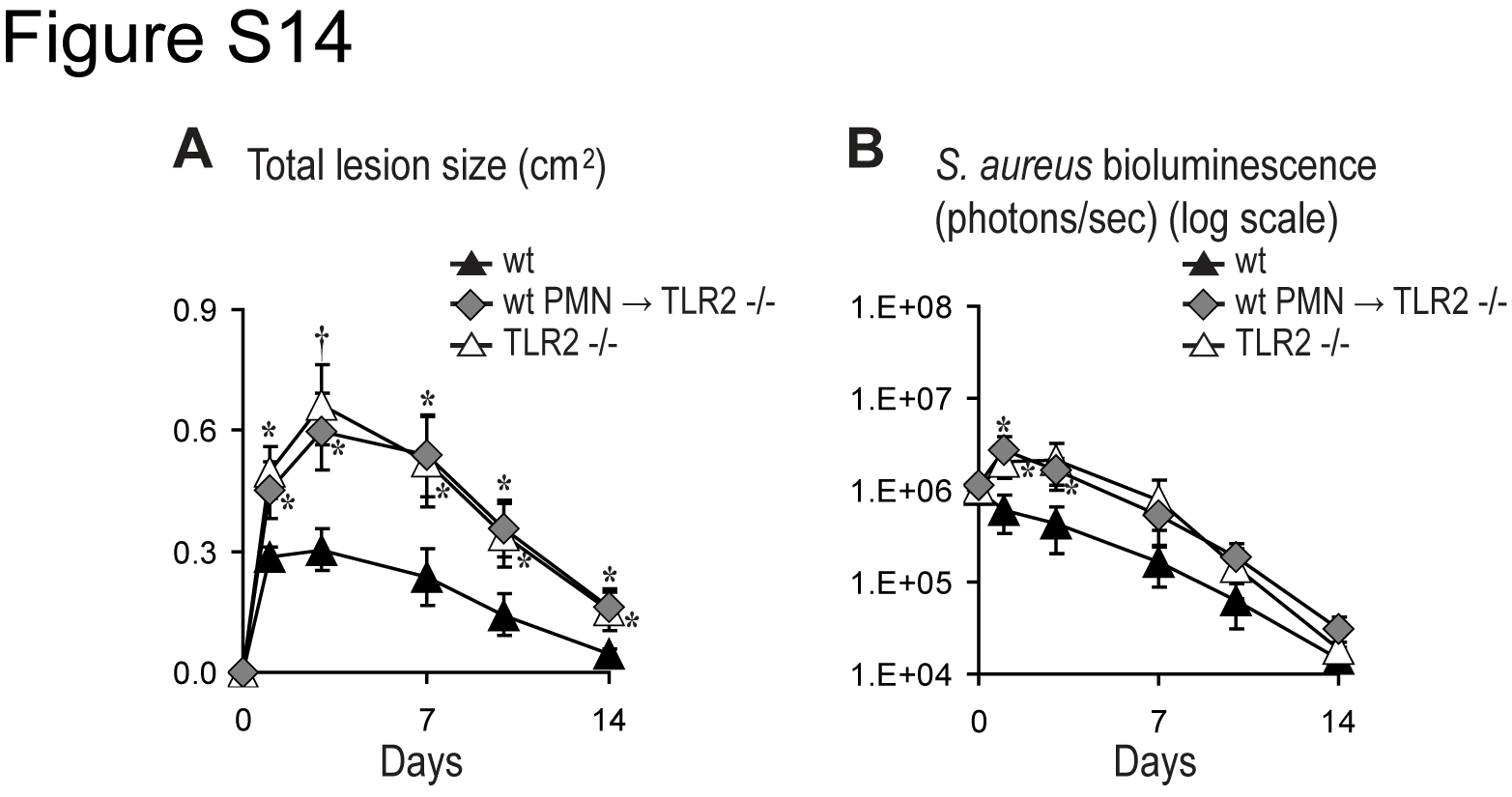

Supplement: Figure S14 — Adoptive transfer of wt neutrophils is not sufficient to rescue TLR2-deficient mice. Neutrophils from wt donor mice were adoptively transferred into TLR2−/− recipient mice. After 2 hrs, these mice and normal wt and TLR2−/− mice were infected intradermally with S. aureus. (A) Mean total lesion size (cm2) ± SEM. (B) In vivo bioluminescence quantified by mean total flux (photons/s) ± SEM (logarithmic scale). Data is representative of at least 4 mice per group. *p<0.05; TLR2−/− mice or TLR2−/− mice with adoptively transferred wt neutrophils versus wt mice (Student's t-test). (TIF) [file ppat.1003047.s014.tif]

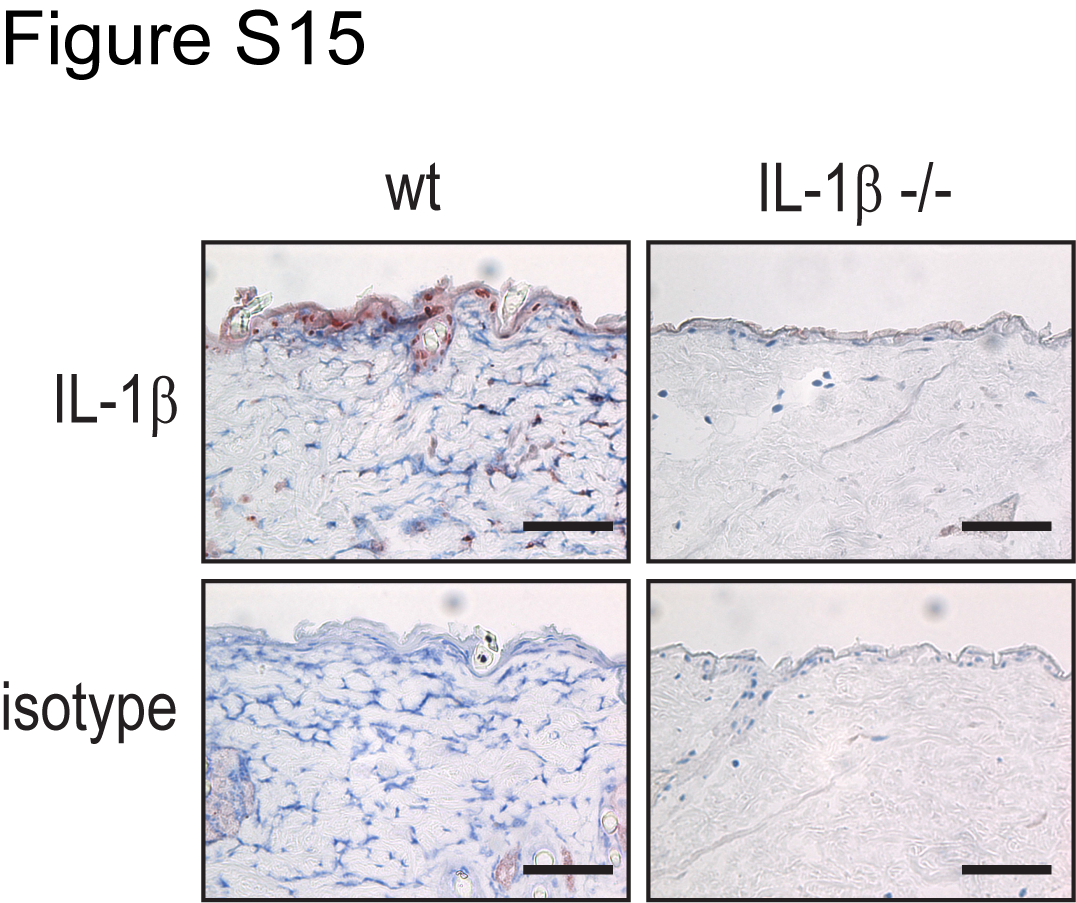

Supplement: Figure S15 — IL-1β protein expression in keratinocytes overlying the S. aureus infection in the dermis. Wt mice were infected intradermally with S. aureus and lesional skin specimens were collected at 24 hrs. Representative photomicrographs of sections labeled with anti-IL-1β mAb or isotype control mAb (immunoperoxidase method) of frozen sections of lesional skin at 1 day after skin inoculation with S. aureus (Scale bars = 50 µm). Data are representative of 3 different wt mice. IL-1β was found to be expressed within some of the epidermal keratinocytes overlying the abscess after skin inoculation with S. aureus. (TIF) [file ppat.1003047.s015.tif]

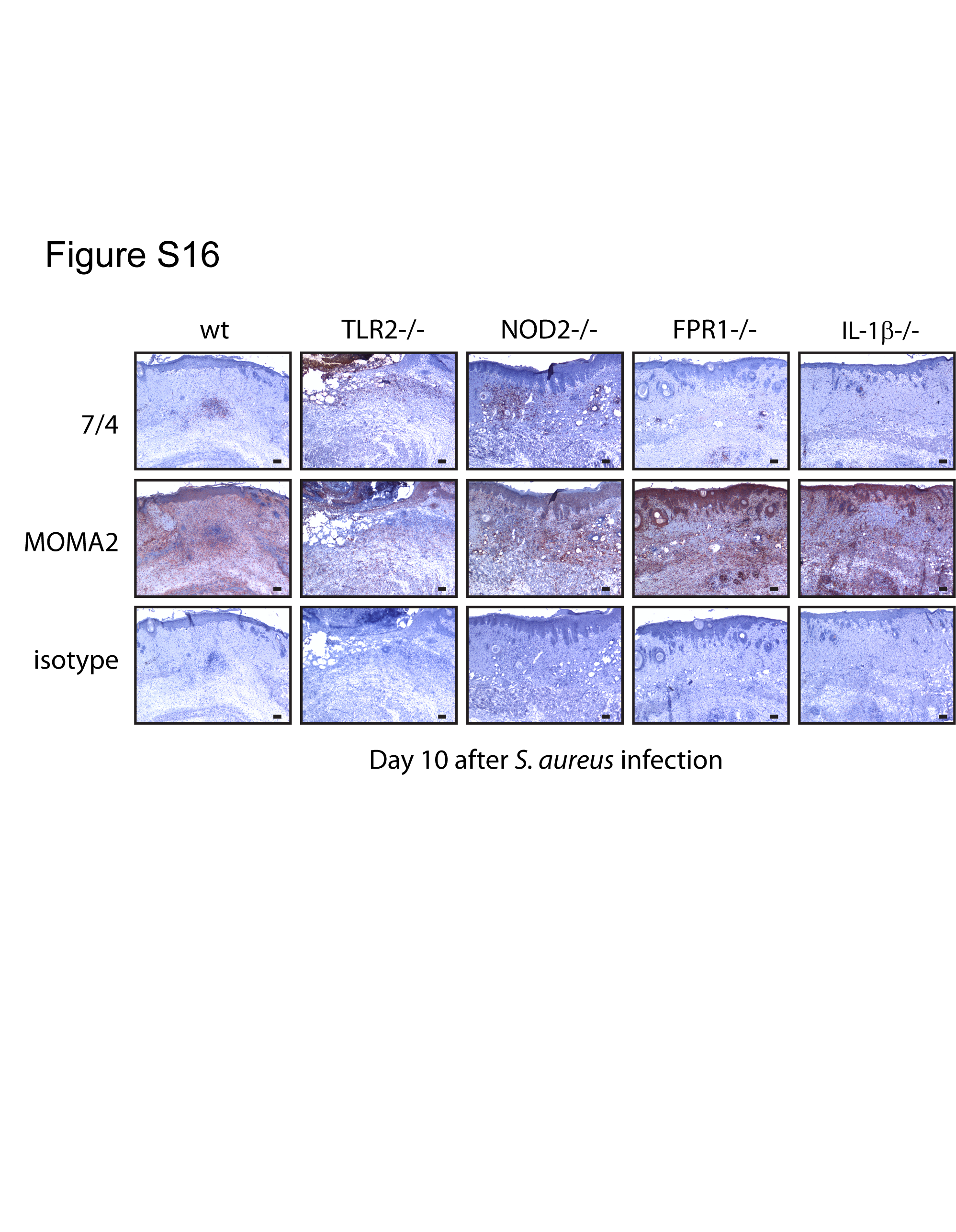

Supplement: Figure S16 — Cellular composition in the S. aureus -infected skin at day 10 after infection. TLR2-, NOD2-, FPR1-, and IL-1β-deficient mice as well as wt mice were inoculated intradermally with S. aureus and lesional skin specimens were collected at 10 days after infection. Representative photomicrographs of sections labeled with anti-7/4 (neutrophils) or anti-MOMA2 (monocytes/macrophages) or isotype control mAb (immunoperoxidase method) of frozen sections lesional skin at 10 days after skin inoculation with S. aureus (Scale bars = 100 µm). Data is representative of 3 mice per group. The cellular composition of 7/4+ neutrophils and MOMA2+ monocytes/macrophages on day 10 in TLR2-, NOD2-, FPR1-deficient mice after infection was similar to the cellular composition in wt mice whereas IL-1β-deficient mice had a paucity of 7/4+ neutrophils at this time point. (TIF) [file ppat.1003047.s016.tif]

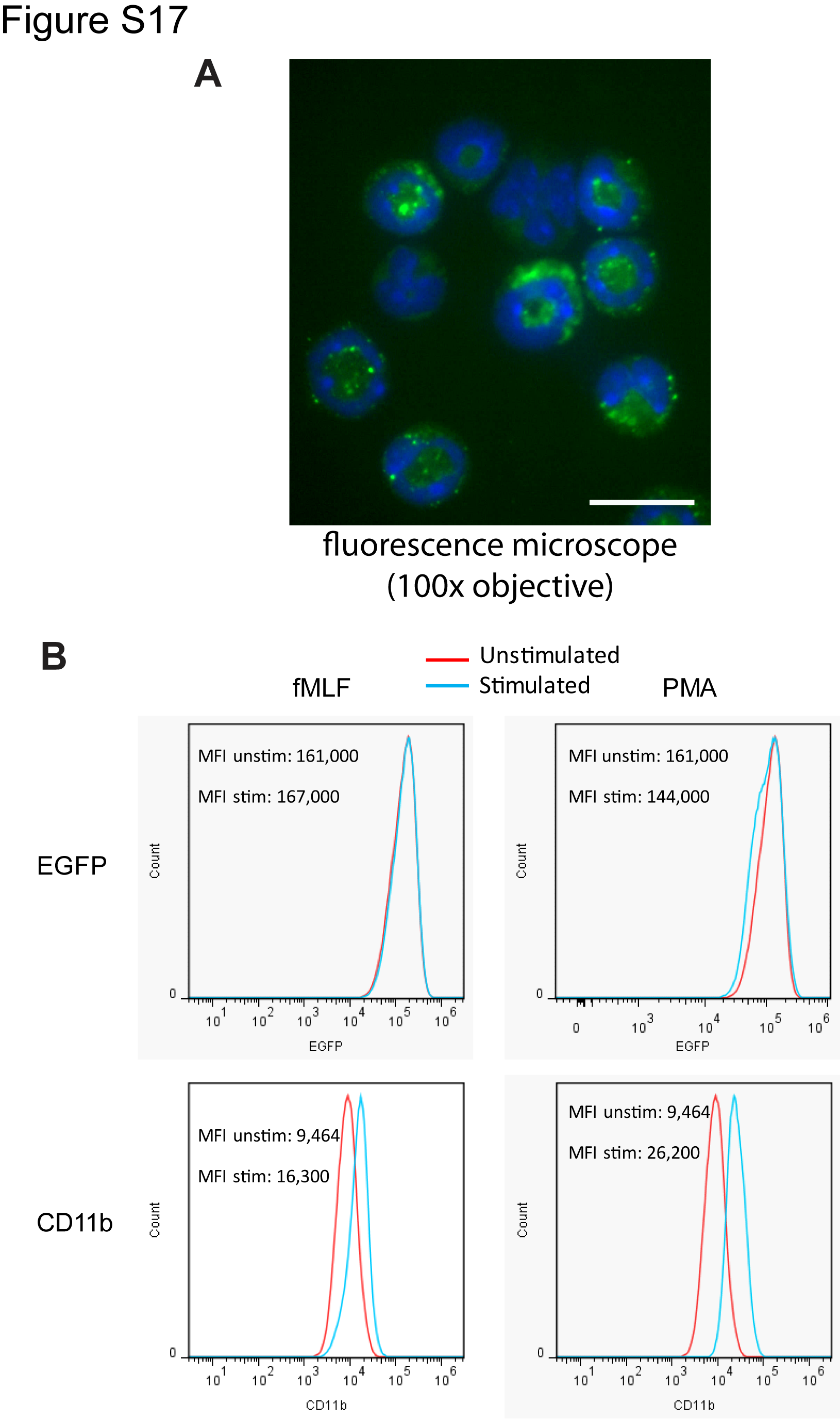

Supplement: Figure S17 — Localization of EGFP in neutrophils from LysEGFP mice. (A) Neutrophils from LysEGFP mice labeled with Hoescht 33342 counterstain, mounted on microscope slides and imaged with an Olympus BX61 fluorescence microscope (100× objective). Green = EGFP and Blue = nucleus. Scale bar = 10 µm. (B) Mouse neutrophils enriched from bone marrow of LysEGFP mice were left unstimulated or stimulated with fMLF or PMA and EGFP fluorescent signals and CD11b expression (as an positive marker for neutrophil activation) were evaluated using flow cytometry. Neutrophils were first gated on forward and side scatter and mature neutrophils were then identified by high Ly6G+ expression. Mean fluorescence intensity (MFI) of EGFP and CD11b on neutrophils is indicated for unstimulated and stimulated neutrophils. Data are representative from 3 different LysEGFP mice. (TIF) [file ppat.1003047.s017.tif]
